# Distinct cellular mechanisms underlie ON orientation selectivity in the mouse retina

**DOI:** 10.1016/j.isci.2026.118130

**Published:** 2026-11-06

**Authors:** Xu Han, Shigang He, Le Sun

**Affiliations:** 1 Beijing Institute of Brain Disorders, Laboratory of Brain Disorders, Ministry of Science and Technology, Collaborative Innovation Center for Brain Disorders, Capital Medical University, Beijing 100069, China; 2 Kaixianghongkang Biotechnology Co., Ltd., Beijing, China; 3 School of Biomedical Engineering, Institute of Natural Sciences and Bio-X Institute, Shanghai Jiao Tong University, Shanghai 200240, China; 4 State Key Laboratory of Brain and Cognitive Science, Institute of Biophysics, Chinese Academy of Sciences, Beijing 100101, China

**Keywords:** orientation selectivity, ON orientation-selective retinal ganglion cells, spiking amacrine cells, GABAergic inhibition, retinal circuits

## Abstract

Orientation selectivity (OS) is a fundamental visual computation. We characterized three morphophysiologically distinct ON orientation-selective ganglion cell (OSGC) populations in the mouse retina: S345 OSGCs and two less frequently recorded groups, putative S13 and S15 OSGCs. Mechanistic analyses focused on S345 OSGCs, which exhibited robust orientation tuning and continuous dendritic stratification across inner plexiform layer strata S3–S5. Their OS depended on a tetrodotoxin (TTX)-sensitive GABAergic inhibitory surround. TTX abolished orientation tuning, whereas 2,3-dihydroxy-6-nitro-7-sulfamoylbenzo[f]quinoxaline (NBQX) reduced orientation-dependent inhibition and eliminated OS, implicating AMPA/kainate receptor-dependent recruitment of spiking inhibitory circuits. Physiologically identified spiking orientation-selective amacrine cells exhibited asymmetric inward-current responses, with orientation bias persisting in TTX. These findings expand the known diversity of ON orientation-selective retinal neurons, suggest distinct patterns of excitation and inhibition underlying their orientation signals, and identify a spiking GABAergic inhibitory mechanism shaping OS in S345 OSGCs.

## Highlights

• Recorded ON OSGCs fall into three morphophysiological groups
• S345 OSGC dendrites stratify continuously across IPL strata S3–S5
• S345 OS requires spiking GABAergic surround inhibition and AMPA/kainate signaling
• Recorded spiking amacrine cells show inward-current orientation bias in TTX

## Graphical abstract

**Figure:**
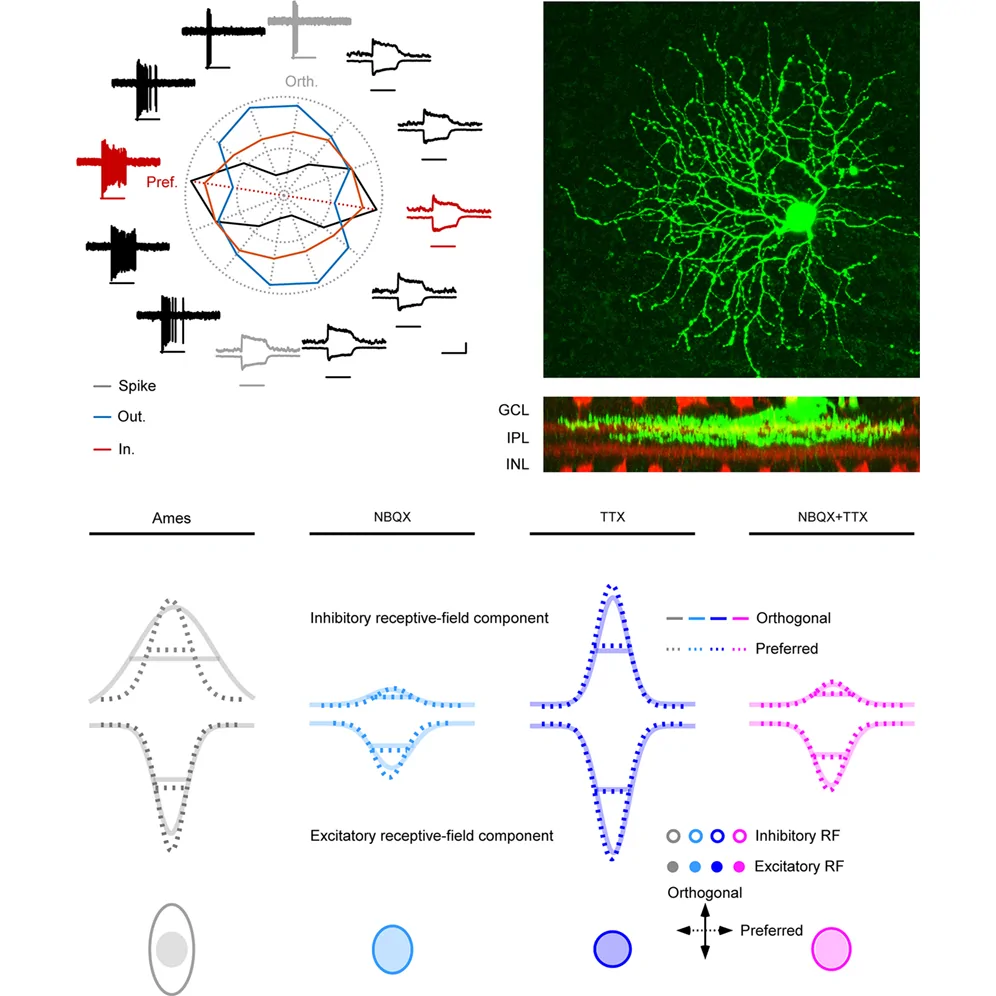

## Teaser

Biological sciences; Natural sciences; Neuroscience

## Introduction

Orientation selectivity (OS)—the preferential response of a neuron to stimuli aligned at particular angles within its receptive field (RF)—is a foundational computation of the visual system and contributes to diverse perceptual and behavioral functions.^1^^,^^2^^,^^3^ OS was first discovered by Hubel and Wiesel in the cat primary visual cortex, where they proposed a feedforward scheme: aligned thalamic (lateral geniculate nucleus [LGN]) inputs with center-surround RFs converge onto a cortical neuron to endow it with OS.^4^ Subsequent work established that intracortical inhibition also shapes OS, complementing feedforward excitation.^1^^,^^5^ Beyond the cortex, OS has been observed in mouse LGN neurons, implicating modulation by retinal ganglion cell (RGC) inputs.^6^^,^^7^^,^^8^ The superior colliculus (SC)—another major retinal target—likewise contains OS neurons,^9^ and mounting evidence suggests that at least part of this tuning is inherited directly from the retina.^10^

Retinal OS was described decades ago in rabbits. Levick identified OS RGCs (OSGCs) comprising ON and OFF center types with preferences clustered along vertical or horizontal axes, and argued that irregular dendritic shape per se is not decisive; instead, anisotropic surround inhibition—rather than asymmetric excitation—underlies the computation.^11^ Pharmacology pointed to a dominant GABAergic contribution to this surround.^12^ In rabbits, ON, OFF, and ON-OFF OSGCs have been reported with distinct dendritic morphologies and stratification profiles,^13^^,^^14^ and subtypes preferring different orientations rely on divergent presynaptic mechanisms.^15^ Orientation-selective and orientation-biased amacrine cells—including polyaxonal amacrine cells—provide candidate circuit elements for these pathways.^14^^,^^16^^,^^17^ In mouse retina, OFF OSGCs^18^ and a bistratified ON OSGC^19^ have been described, but their mechanisms and pharmacology differ from those in rabbits. Given the extensive morphological and functional diversity of mouse RGCs,^20^^,^^21^^,^^22^^,^^23^ the diversity and circuit logic of mouse OSGCs remain incompletely defined. In particular, it remains unclear how many ON OSGC types contribute to retinal OS output, whether these cell types differ in their sensitivity to oriented stimuli, and whether ON retinal OS is implemented through a common circuit motif or through distinct cellular mechanisms.

Here, we characterize three morphophysiologically distinguishable ON orientation-selective RGC groups in the mouse retina. These groups differ in dendritic field size, inner plexiform layer (IPL) stratification, and responses to bars of different lengths, suggesting differences in their spatial integration properties. We then focus on one experimentally tractable group, an ON-sustained OSGC whose dendrites stratify continuously in IPL S3–S5, including partial arborization in S4, yet lack direction selectivity, arguing against canonical starburst amacrine cell (SAC)-mediated direction-selective circuitry. Using this S3/4/5-stratifying OSGC (S345 OSGC) as a model, we show that its OS arises from a RF organization consisting of a symmetric excitatory center together with an inhibitory surround elongated along the orthogonal axis. This surround is mediated by tetrodotoxin (TTX)-sensitive GABAergic input, indicating a spiking-dependent presynaptic inhibitory source. In parallel, we recorded physiologically identified spiking ON orientation-selective amacrine cells (OSACs) that exhibited asymmetric inward-current responses, with the orientation bias of these currents persisting in TTX. The overlap in stratification between OSAC processes and S345 OSGC dendrites suggests possible participation of these amacrine cells in ON retinal orientation-selective circuitry, although a direct synaptic connection to S345 OSGCs was not demonstrated.

Together, these findings expand the known diversity of ON orientation-selective retinal neurons and suggest that distinct patterns of excitation and inhibition contribute to their orientation signals. They identify a prominent role for TTX-sensitive GABAergic surround inhibition in S345 OSGCs and provide a basis for investigating how orientation information is processed across different retinal cell populations.

## Results

### Morphophysiological characterization of three groups of ON orientation-selective RGCs in the mouse retina

We assayed OS in mouse RGCs using flashing bar stimuli and quantified spike rates across orientations. The tested orientation closest to the peak of the fitted von Mises curve was defined as the preferred orientation, and the perpendicular orientation as the orthogonal orientation (Figures 1A, 1E, S1A, and S1E). Cells with a spike-based orientation bias index (OBI) >0.3 were classified as orientation-selective ganglion cells (OSGCs; see STAR Methods). Using this criterion together with morphological features, we found that the recorded OSGCs could be assigned to three morphophysiological populations, suggesting that ON orientation-selective signals in the mouse retina are not restricted to a single canonical output channel.

**Figure 1:**
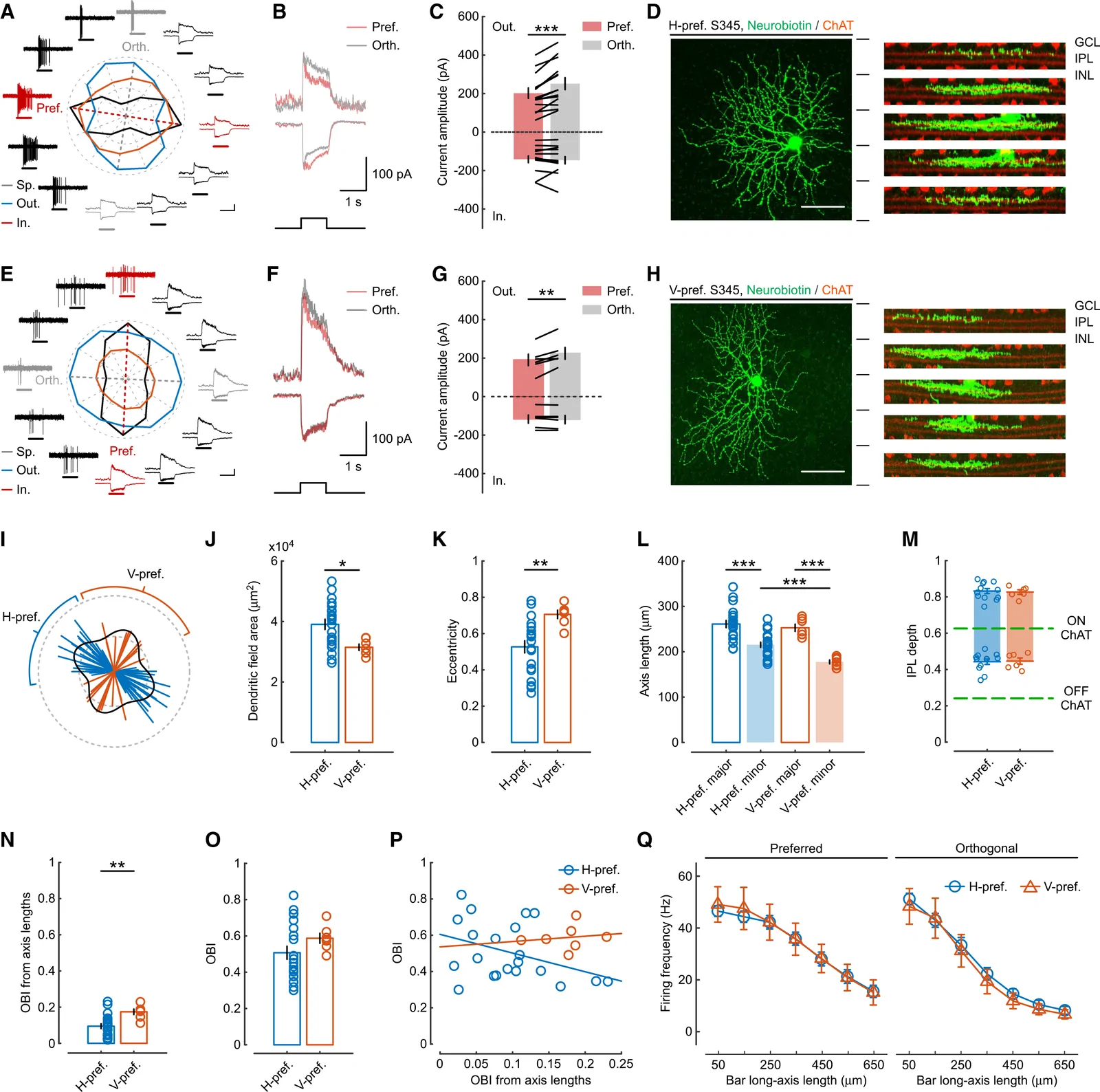
Physiological and morphological characterization of S345 ON OSGCs in the mouse retina (A and E) Polar plots and representative responses of horizontal-preferring (A, H-pref.) and vertical-preferring (E, V-pref.) S345 OSGCs to bar stimuli presented at different orientations. Polar plots show spike responses (black), outward currents (blue), and inward currents (orange). Spike responses were normalized to the response at the preferred orientation. The absolute mean amplitudes of both outward and inward currents were normalized to the absolute mean outward-current amplitude evoked at the orthogonal orientation. Representative spike trains are arranged along the left semicircle according to stimulus orientation, whereas representative outward- and inward-current traces are arranged along the right semicircle. Horizontal lines below the traces indicate the 1-s stimulus duration. Red traces indicate responses to bars presented at the preferred orientation, whereas gray traces indicate responses to bars presented at the orthogonal orientation, perpendicular to the preferred axis. Scale bars: 1 s (horizontal) and 100 pA (vertical). (B and F) Light-evoked inward and outward currents recorded from H-pref. (B) and V-pref. (F) S345 OSGCs in response to bars presented along the preferred (red) and orthogonal (gray) orientations. Inward and outward currents were recorded under whole-cell voltage clamp at holding potentials of −65 and 20 mV, respectively. (C and G) Average amplitudes of light-evoked outward and inward currents in H-pref. (C) and V-pref. (G) S345 OSGCs in response to 1-s bar stimuli presented along the preferred (red) and orthogonal (gray) orientations. In (C), *n* = 14 cells from 14 mice, *p* = 3.4 × 10^−5^ and 0.4428 for outward and inward currents, respectively. In (G), *n* = 6 cells from 6 mice, *p* = 0.0075 and 0.2183 for outward and inward currents, respectively. Two-sided paired *t* test. (D and H) Morphology of H-pref. (D) and V-pref. (H) S345 OSGCs (left, green) and their dendritic stratification within the inner plexiform layer (right, green). ChAT immunostaining (red) marks the ON and OFF bands. Scale bars, 50 μm. (I) Preferred orientations and orientation bias indices (OBIs) of recorded S345 OSGCs. Each cell is represented along its preferred orientation axis, with radial length proportional to its OBI. H-pref. cells (*n* = 29 cells from 29 mice) are shown in blue, and V-pref. cells (*n* = 10 cells from 10 mice) are shown in orange. The black curve represents a least-squares fit of OBI values using a baseline plus two axial von Mises functions. The fitted H-pref. and V-pref. components had full widths at half maximum (FWHMs) of 72.2° and 85.8°, respectively. (J) Comparison of dendritic field areas between H-pref. and V-pref. S345 OSGCs. For H-pref. and V-pref. S345 OSGCs, *n* = 21 and 7 cells from 21 and 7 mice, respectively; *p* = 0.0158, two-sided unpaired *t* test. (K) Comparison of eccentricity of ellipse-fitted dendritic fields between H-pref. and V-pref. S345 OSGCs. For H-pref. and V-pref. S345 OSGCs, *n* = 21 and 7 cells from 21 and 7 mice, respectively; *p* = 0.0049, two-sided unpaired *t* test. (L) Comparison of the major and minor axes of ellipse-fitted dendritic fields between H-pref. and V-pref. S345 OSGCs. For H-pref. and V-pref. S345 OSGCs, *n* = 21 and 7 cells from 21 and 7 mice, respectively; *p* = 1.6 × 10^−6^ for the major versus minor axes in H-pref. S345 OSGCs, two-sided paired *t* test; *p* = 3.2 × 10^−5^ for the major versus minor axes in V-pref. S345 OSGCs, two-sided paired *t* test; *p* = 0.5639 for the major axes between H-pref. and V-pref. S345 OSGCs, two-sided unpaired *t* test, and *p* = 8.3 × 10^−4^ for the minor axes between H-pref. and V-pref. S345 OSGCs, two-sided unpaired *t* test. (M) Stratification profiles of dendritic arbors of H-pref. and V-pref. S345 OSGCs within the IPL. The upper and lower bounds indicate the extent of each cell’s dendritic arbor along the IPL depth axis. For H-pref. and V-pref. S345 OSGCs, *n* = 12 and 6 cells from 12 and 6 mice, respectively. (N) Morphology-based OBI calculated from the major and minor axes of dendritic fields. For H-pref. and V-pref. S345 OSGCs, *n* = 21 and 7 cells from 21 and 7 mice, respectively; *p* = 0.0026, two-sided unpaired *t* test. (O) Response-based OBI calculated from light-evoked spiking responses. For H-pref. and V-pref. S345 OSGCs, *n* = 21 and 7 cells from 21 and 7 mice, respectively; *p* = 0.2182, two-sided unpaired *t* test. (P) Relationship between morphology-based OBI and response-based OBI in S345 OSGCs. Morphology-based OBI was calculated from the lengths of the major and minor axes of dendritic fields, whereas response-based OBI was calculated from light-evoked spiking responses. Each symbol represents one cell. Blue and orange circles indicate H-pref. and V-pref. S345 OSGCs, respectively. Lines represent ordinary least-squares linear fits with an intercept. For H-pref. S345 OSGCs, *n* = 21 cells from 21 mice, R^2^ = 0.1442, and *p* = 0.0896; for V-pref. S345 OSGCs, *n* = 7 cells from 7 mice, R^2^ = 0.0257, and *p* = 0.7313. *p* values were calculated using two-sided Pearson correlation tests. (Q) Averaged spiking responses of H-pref. and V-pref. S345 OSGCs to elongated bar stimuli presented along the preferred (left) and orthogonal (right) orientations. For H-pref. and V-pref. S345 OSGCs, *n* = 29 and 10 cells from 29 and 10 mice, respectively. ∗*p* < 0.05, ∗∗*p* < 0.01, ∗∗∗*p* < 0.001. Data are shown as mean ± SEM.

Cells in the most frequently recorded group showed sustained ON spiking during light stimulation and preferred either near-horizontal orientations (horizontal-preferring [H-pref.]; Figure 1A) or near-vertical orientations (vertical-preferring [V-pref.]; Figure 1E). Voltage-clamp recordings revealed sustained light-evoked inward and outward currents in response to both preferred and orthogonal bar stimuli (Figures 1A, 1B, 1E, and 1F). In both H-pref. and V-pref. S345 OSGCs, bars presented at the orthogonal orientation evoked larger outward currents than those presented at the preferred orientation, whereas inward currents did not differ significantly between orientations (Figures 1C and 1G). Across S345 OSGCs, outward-current-based OBI was significantly associated with spike-based OBI in a linear regression analysis (R^2^ = 0.3931, *p* = 0.0041; Figure S2A), indicating that cells with greater orientation-dependent differences in outward current tended to exhibit stronger spike-based orientation tuning. Mean spike-based OBI remained broadly stable across the five repeated presentations of the oriented-bar stimuli, with no significant effect of stimulus repetition in either subgroup (Figure S2B). Because currents were recorded at only two holding potentials, without determining synaptic reversal potentials in individual cells, we did not estimate excitatory or inhibitory conductances or E/I conductance ratios. These cells had intermediate-sized, densely branched dendritic arbors distributed continuously across IPL strata S3–S5, with only occasional sparse dendritic terminals extending into S1 (Figures 1D, 1H, and S3A–S3D), morphologically resembling B3 inner cells.^20^ We therefore refer to them as S345 OSGCs.

Unlike typical ON-OFF direction-selective ganglion cells (DSGCs; Figures S4A and S4B), S345 OSGCs lacked direction selectivity despite partially arborizing within the S4 sublamina (Figures S4C–S4E), arguing against their classification as conventional ON-OFF DSGCs. S345 OSGCs were assigned to H-pref. and V-pref. groups according to their proximity to the two fitted orientation axes, located near 153.2° and 70.1°, respectively (Figure 1I). V-pref. S345 OSGCs had smaller dendritic field areas than H-pref. cells (Figure 1J). Ellipse fitting further showed that V-pref. S345 OSGCs had higher dendritic field eccentricity and more elongated dendritic arbors (Figures 1K and 1L), whereas the two subgroups showed similar dendritic stratification within the IPL (Figure 1M). We next calculated a morphology-based OBI from the major and minor axes of dendritic fields (Figure 1N) and spike-based OBI from responses to preferred and orthogonal bars (Figure 1O). No significant linear correlation was detected between these two OBI measures in either H-pref. or V-pref. S345 OSGCs (Figure 1P), suggesting that dendritic-field elongation alone may be insufficient to explain their OS. Finally, when elongated bar stimuli of increasing length were presented along the preferred or orthogonal orientation, H-pref. and V-pref. S345 OSGCs showed similar length-dependent response profiles (Figure 1Q), consistent with a contribution of RF surround mechanisms to their orientation-selective responses. Because the numbers of H-pref. and V-pref. cells in the subsequent voltage-clamp and pharmacological experiments were small and unequal, these mechanistic datasets were not further subdivided for subgroup-level statistical comparisons.

The second population showed a brief ON response that did not persist throughout the 1-s bar stimulus (Figure S1A). Voltage-clamp recordings revealed comparable outward currents between preferred and orthogonal orientations, whereas inward currents showed a nonsignificant tendency to be larger for bars presented along the preferred orientation (Figures S1B and S1C). These cells had small, compact dendritic arbors stratifying in IPL sublaminae S1 and S3 (Figures S1D and S1M), resembling the B2 type.^20^ Because only 6 cells were recorded, their classification remains tentative, and we therefore refer to them as putative S13 OSGCs. Their compact morphology and transient spiking pattern suggest a relatively spatially restricted mode of visual integration compared with S345 OSGCs.

The third population showed sustained spiking during light stimulation, often with activity persisting after light offset (Figure S1E). Consistent with this response pattern, voltage-clamp recordings revealed slowly decaying inward currents, whereas outward currents differed between preferred and orthogonal orientations (Figures S1F and S1G). Morphologically, these cells were bistratified, with broad dendritic arbors stratifying in S1 and near the S4/S5 border of the IPL (Figures S1H and S1M). Because their bistratified morphology and some response properties (Figure S1I) partially resemble those of previously reported bistratified ON OSGCs,^19^ we provisionally refer to them as S15 OSGCs. Their broad dendritic fields and sustained firing suggest a mode of spatial integration distinct from those of S345 and putative S13 OSGCs.

S15 OSGCs had larger dendritic field areas than putative S13 OSGCs (Figure S1J). Ellipse-fitted dendritic fields of putative S13 and S15 OSGCs showed similar eccentricity values (Figure S1K) and anisotropic geometry in both populations (Figure S1L). Similarly, morphology-based OBI showed no significant association with response-based OBI in putative S13 or S15 OSGCs (Figures S1N–S1P), further indicating that dendritic-field geometry alone may not fully explain orientation tuning. Responses to elongated bars further revealed distinct length-dependent profiles: putative S13 OSGCs showed reduced spiking to longer bars despite OBI values above 0.3, whereas S15 OSGCs maintained stronger preferred-orientation responses as bar length increased (Figure S1Q).

Together, these results suggest that ON orientation-selective signals are represented by multiple morphophysiological OSGC populations. Because S345 OSGCs were the most frequently recorded group and exhibited robust orientation tuning, we focused subsequent mechanistic analyses on this population.

### Action potential-dependent peripheral inhibition contributes to orientation tuning in S345 OSGCs

OS in RGCs can arise through multiple mechanisms, including dendritic asymmetry, asymmetric excitation, and synaptic inhibition. However, the morphology-based OBI was not significantly correlated with the spike-based OBI in S345 OSGCs, suggesting that the magnitude of dendritic asymmetry alone is unlikely to account for the strength of their OS. We therefore examined whether spatially extended, orientation-dependent inhibition contributes to OS in S345 OSGCs. Because long-range surround signals in the retina are often mediated by wide-field spiking amacrine cells (WACs),^14^^,^^16^^,^^24^ we tested whether action potential-dependent peripheral inhibition contributes to orientation tuning.

We first mapped each cell’s RF center using random flicker stimuli (50-μm spots) and registered spike responses according to spot position (Figures S5A and S5B). Oriented-bar stimuli presented at multiple positions across the RF center yielded consistent orientation preference (Figure S5C), indicating that asymmetric inhibitory input from the periphery contributes to shaping OS in S345 OSGCs. To further probe spatial organization, we presented 100 × 100 μm flashing squares at 100-μm intervals along the preferred and orthogonal axes, centered on the RF center (Figure 2A). Under voltage clamp, S345 OSGCs displayed a symmetric excitatory center (inward currents) ∼300 μm in diameter along both axes (Figures 2B, S6A, and S6B). In contrast, inhibitory currents were highly anisotropic: inhibition along the preferred axis slightly exceeded the excitatory center (Figures 2B and S6B, left, gray line), whereas inhibition along the orthogonal axis extended much farther, with peripheral inhibitory input still detectable at locations up to 600 μm from the RF center in some cases (Figures 2B and S6B, right, gray line).

**Figure 2:**
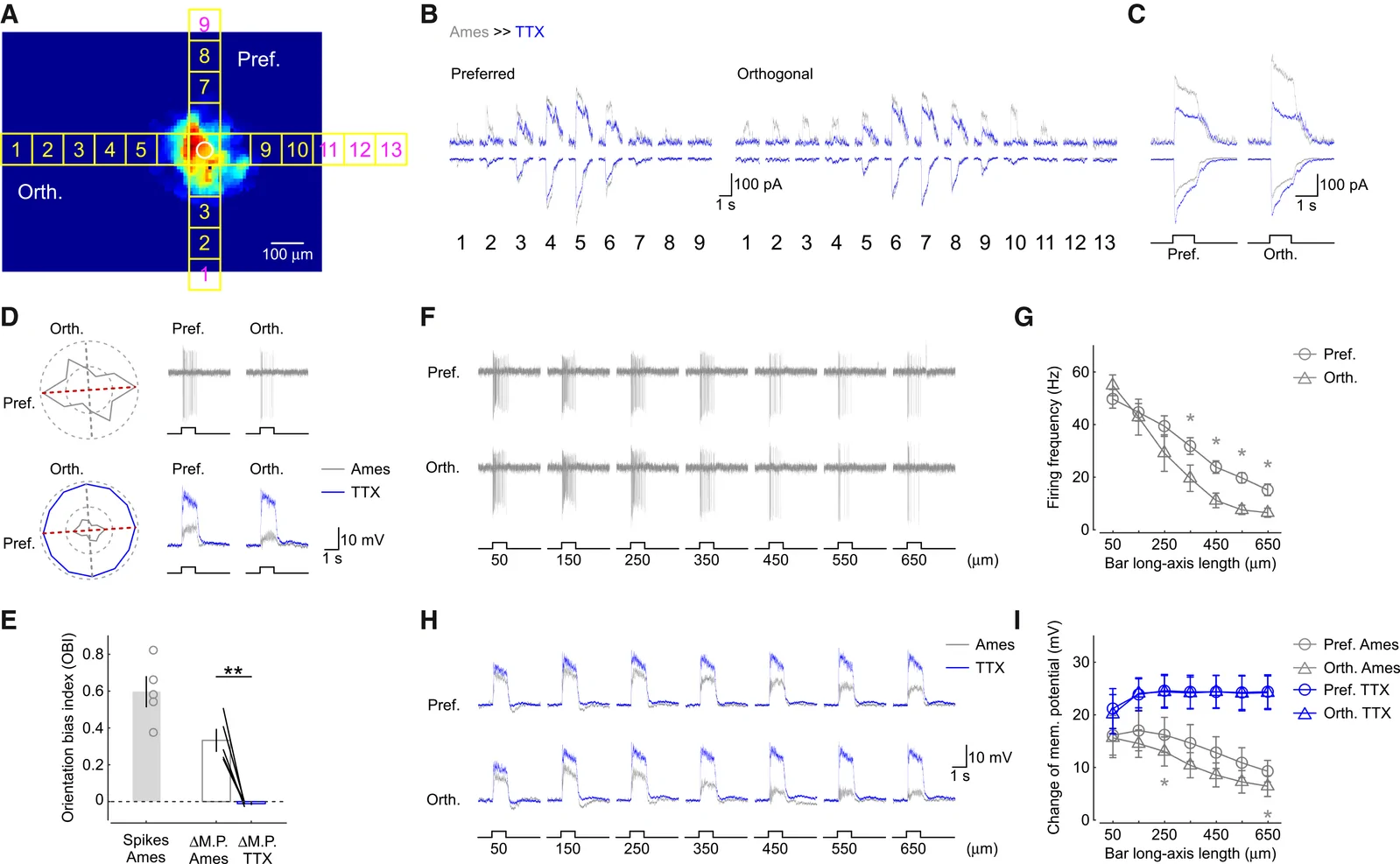
Orthogonal-biased asymmetric TTX-sensitive inhibition shapes S345 OSGC tuning (A) Schematic of receptive field mapping in S345 OSGCs. The excitatory receptive field center was identified, and stimuli were delivered along the preferred (pref.) and orthogonal (orth.) axes in 100-μm steps. (B) Representative voltage-clamp recordings of S345 OSGCs in response to flashing square stimuli delivered along the pref. and orth. axes under control conditions (Ames) and in the presence of TTX (100 nM). (C) Representative voltage-clamp responses of S345 OSGCs to bar stimuli presented along the pref. and orth. axes under control conditions and in TTX. (D) Spiking responses (top) and membrane potential changes (bottom) of an S345 OSGC to bar stimuli at different orientations before and after TTX application. (E) Changes in membrane potential-based orientation bias index (OBI) of S345 OSGCs following TTX application (*p* = 0.0030, two-sided paired *t* test). Spike-based OBI under control conditions (Ames) is shown for reference. All measurements were obtained from the same 5 cells from 5 mice. (F and G) Representative spiking responses (F) and averaged responses across cells (G) of S345 OSGCs to elongated bar stimuli of different lengths presented along the pref. and orth. axes. In (G), *n* = 4 cells from 4 mice; *p* = 0.0395, 0.0262, 0.0170, and 0.0136; two-sided paired *t* test. (H and I) Representative current-clamp responses (H) and averaged responses across cells (I) of S345 OSGCs to elongated bar stimuli of varying lengths presented along the pref. and orth. axes before and after TTX application. In (I), *n* = 4 cells from 4 mice under both Ames and TTX conditions; *p* = 0.0365 and 0.0282 (pref. Ames vs. orth. Ames); two-sided paired *t* test. ∗*p* < 0.05, ∗∗*p* < 0.01. Data are shown as mean ± SEM.

Application of TTX, which blocks action potentials, left excitation largely unchanged but selectively reduced inhibition at the RF periphery along the orthogonal axis while modestly affecting inhibition within the center (Figures 2B and S6C). Consistently, during oriented-bar stimulation, TTX abolished the orientation-dependent difference in inhibitory input (Figures 2C, 3F, and 3G). These results identify TTX-sensitive distal inhibition preferentially aligned with the orthogonal axis as a key determinant of S345 OSGC orientation tuning.

**Figure 3:**
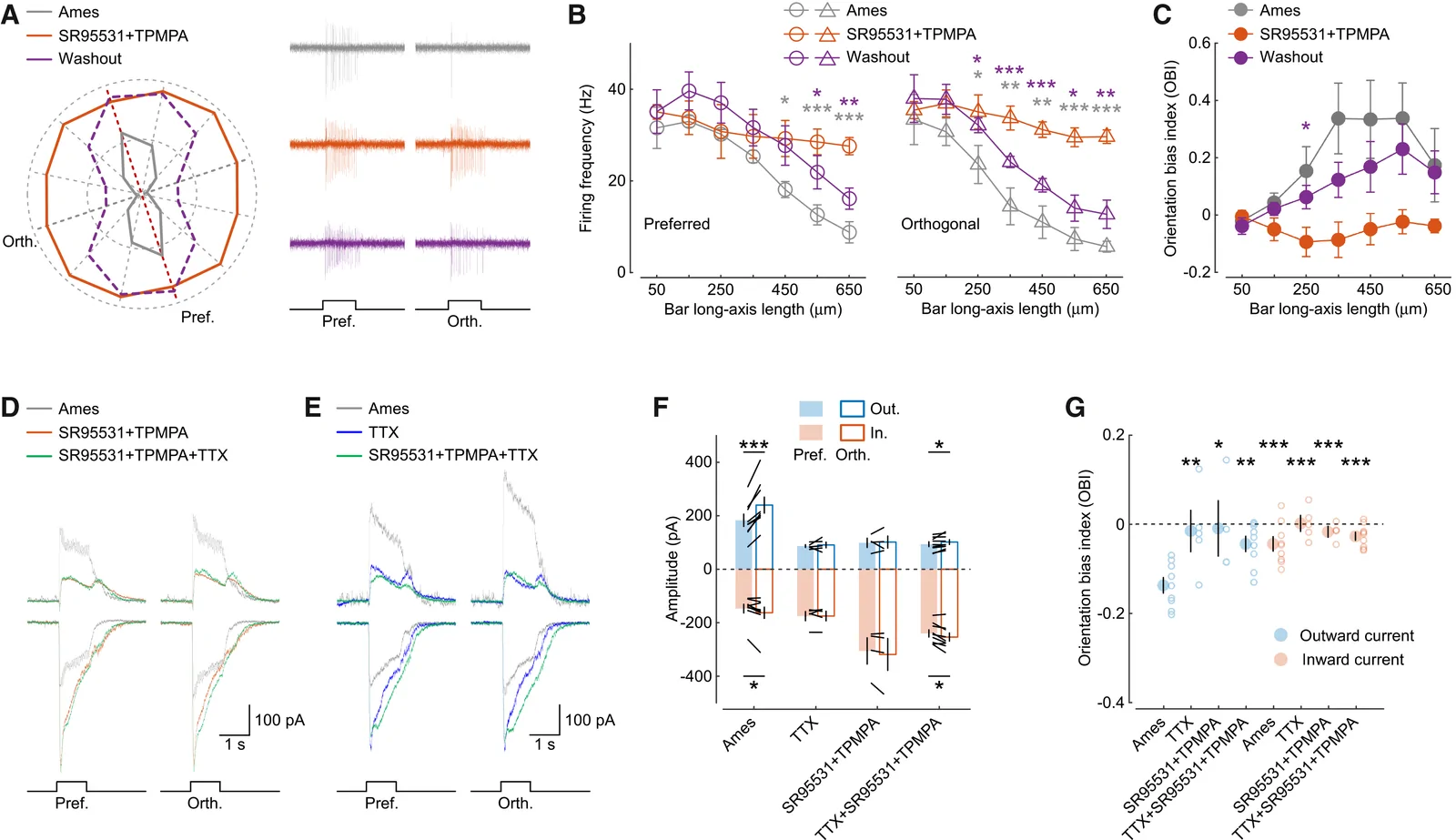
S345 OSGCs receive TTX-sensitive GABAergic inhibition (A) Polar plots showing spiking responses of a representative S345 OSGC to bar stimuli of different orientations under control conditions (Ames), during application of SR95531 (5 μM) + TPMPA (50 μM), and after washout (left). Representative spike trains evoked by bars along the preferred and orthogonal orientations are shown on the right. (B and C) (B) Averaged spiking responses of S345 OSGCs to elongated bar stimuli presented along the preferred (left) or orthogonal (right) orientations under control (Ames), SR95531 + TPMPA, and washout conditions. For the preferred orientation: gray asterisks, Ames vs. SR95531 + TPMPA, *p* = 0.0231, 0.00096, 0.00030; purple asterisks, washout vs. SR95531 + TPMPA, *p* = 0.0491, 0.0024. For the orthogonal orientation: gray asterisks, Ames vs. SR95531 + TPMPA, *p* = 0.0317, 0.0045, 0.0013, 0.00046, 0.000004; purple asterisks, washout vs. SR95531 + TPMPA, *p* = 0.0363, 0.00088, 0.00023, 0.0191, 0.0067. (C) Orientation bias index (OBI) of S345 OSGCs in response to bars of different lengths under Ames, SR95531 + TPMPA, and washout conditions. Purple asterisk, washout vs. SR95531 + TPMPA, *p* = 0.0452. For (B) and (C), Ames and SR95531 + TPMPA measurements were obtained from the same 5 cells from 5 mice, with washout data available for 4 of these cells. Comparisons were performed using two-sided paired t-tests, with 5 pairs for Ames vs. SR95531 + TPMPA and 4 pairs for washout vs. SR95531 + TPMPA. (D) Representative voltage-clamp recordings from an S345 OSGC in response to bar stimuli along the preferred (Pref.) and orthogonal (Orth.) axes under control (Ames), SR95531 (5 μM) + TPMPA (50 μM), and SR95531 (5 μM) + TPMPA (50 μM) + TTX (100 nM). (E) Representative voltage-clamp recordings under control (Ames), TTX (100 nM), and SR95531 (5 μM) + TPMPA (50 μM) + TTX (100 nM) conditions. (F) Amplitudes of outward and inward currents evoked by bars presented along the preferred and orthogonal orientations under control (Ames), TTX (100 nM), SR95531 + TPMPA, and SR95531 + TPMPA + TTX conditions (*n* = 9, 5, 4, and 9 cells, respectively, from 9 mice). *p* = 0.00019 (Ames outward), 0.0345 (SR95531 + TPMPA + TTX outward), 0.0277 (Ames, inward), 0.0116 (SR95531 + TPMPA + TTX, inward). Two-sided paired *t* test. (G) OBI of mean inward and outward currents evoked by bars presented along the preferred and orthogonal orientations under the same four pharmacological conditions (*n* = 9, 5, 4, and 9 cells, respectively, from 9 mice). OBI values derived from outward currents in Ames were compared with those from other conditions: *p* = 0.0070, 0.0117, 0.0010, 0.00079, 0.00014, 0.0008, and 0.000021. Two-sided unpaired *t* test. ∗*p* < 0.05, ∗∗*p* < 0.01, ∗∗∗*p* < 0.001. Data are shown as mean ± SEM.

We next assessed the impact on spiking and membrane potential. Under control conditions, S345 OSGCs fired more robustly to preferred than to orthogonal bars (Figure 2D top). TTX eliminated this difference (Figure 2D, bottom), and the OBI dropped toward zero (Figure 2E). In current clamp with intracellular QX-314 to block ganglion-cell spiking, subthreshold membrane potentials still exhibited partial orientation tuning (OBI > 0.3; Figure 2E), suggesting that intrinsic spike-dependent nonlinearities of the ganglion cell contribute to optimizing, rather than generating, OS.

Finally, when bar length increased along each axis, spike responses declined with length and showed orientation-dependent differences between preferred and orthogonal axes (Figures 2F and 2G). Membrane-potential responses showed a similar trend, with preferred-orientation bars generally evoking larger depolarizing responses than orthogonal-orientation bars, although significant differences were observed only at some bar lengths (Figures 2H and 2I). In the presence of TTX, this orientation-dependent difference in membrane-potential responses was no longer observed; instead, responses increased with bar length until reaching the estimated dendritic-field edge (∼250 μm diameter) and then saturated. Together, these findings are consistent with a circuit model in which S345 OSGCs receive broadly isotropic excitation within the dendritic field, while action potential-dependent peripheral inhibition contributes to orientation tuning by preferentially suppressing responses along the orthogonal axis.

### The TTX-sensitive inhibitory surround in S345 OSGCs is GABAergic

Previous work in mouse, rabbit, and zebrafish has shown that both GABAergic and glycinergic inhibition contribute to retinal OS.^16^^,^^17^^,^^19^^,^^25^^,^^26^ We next used pharmacological manipulations to identify the transmitters and receptors underlying the TTX-sensitive surround inhibition in OSGCs.

Bath application of the GABA receptor antagonist picrotoxin (100 μM) abolished OS in OSGCs (Figures S7A–S7C), whereas the glycine receptor antagonist strychnine (2 μM) produced only a mild reduction (Figures S7D–S7F). These results implicate GABAergic signaling as a major contributor to OS in S345 OSGCs. Consistently, blockade of GABA_A_ receptors with SR95531 (5 μM) markedly reduced inhibitory inputs evoked by both preferred and orthogonal stimuli, eliminating their orientation difference, while also diminishing excitatory inputs (Figures S7G and S7H). These findings indicate that GABA_A_-mediated inhibition contributes importantly to OS and additionally exerts a disinhibitory control over excitatory drive to OSGCs.

To test the combined contribution of GABA_A_ and GABA_C_ receptors, we applied SR95531 together with ((1,2,5,6-tetrahydropyridin-4-yl)methylphosphinic acid [TPMPA]; 50 μM). Representative recordings showed increased spiking across orientations and reduced orientation tuning during blockade, with partial recovery of tuning after washout (Figure 3A). With increasing bar length, S345 OSGCs showed asymmetric GABAergic suppression: along the orthogonal axis, inhibition curtailed spiking for shorter bars (∼250 μm) than along the preferred axis (Figure 3B). Mean OBI values were closer to zero during blockade (Figure 3C). These observations are consistent with a contribution of GABAergic surround inhibition to OS in S345 OSGCs. We therefore examined how this asymmetric surround was reflected in the underlying synaptic currents.

We next related this surround to spike dependence and quantified its impact on synaptic currents. Bar stimuli along the preferred and orthogonal axes evoked orientation-dependent inhibitory currents with largely isotropic excitation (Figures 3D and 3F). Blockade of GABA_A_/GABA_C_ receptors substantially reduced inhibition, abolished its orientation dependence, and concurrently enhanced excitatory currents (Figures 3D and 3F), suggesting tonic GABAergic suppression of excitatory input. Subsequent addition of TTX after GABA blockade produced no further change (Figures 3D and 3F), indicating that the spike-driven component of surround inhibition operates through GABAergic transmission. Reversing the sequence, TTX alone greatly reduced inhibition, eliminated its orientation dependence, and enhanced—but did not orient—excitation (Figures 3E and 3F). Accordingly, outward currents showed a stronger bias toward the orthogonal orientation under control conditions (Ames), reflected by significantly more negative OBI values than those observed in TTX, SR95531 + TPMPA, or combined TTX + SR95531 + TPMPA conditions (Figure 3G). Although this current bias was modest, its attenuation following TTX or GABA receptor blockade, together with its association with spike-based OBI, supports a functional contribution of orientation-dependent inhibition to spike-based orientation tuning.

Together, these results indicate that action potential-dependent GABA_A_/GABA_C_ surround inhibition contributes importantly to the orientation tuning in S345 OSGCs, whereas excitatory inputs remain largely isotropic. Although the inhibitory current bias was modest in magnitude, its attenuation following TTX or GABA receptor blockade, together with its correlation with spike-based OBI, supports its functional contribution to spike-level OS.

### AMPA/kainate receptor activation recruits the spike-dependent inhibitory surround in S345 OSGCs

WACs are prevalent in the retina,^27^^,^^28^ and a subset exhibits OS,^14^^,^^16^ making them plausible sources of the asymmetric inhibitory surround impinging on OSGCs. Many WACs display AMPA receptor-mediated sustained ON responses, suggesting a role for glutamatergic drive in generating OS.

We therefore blocked AMPA/kainate receptors with NBQX to probe their contribution. Under NBQX, OSGC spike responses increased overall to oriented bars but lost orientation dependence (Figure 4A). Compared with oriented-bar stimulation, a light spot centered on the OSGC RF evoked spiking that was strongly attenuated by NBQX, yielding a significant reduction in firing rate (Figures 4B and 4C), consistent with a disproportionate NBQX effect on the RF center versus the surround. In voltage-clamp recordings, NBQX reduced the ON-phase inhibitory currents evoked by bar stimuli and abolished the difference in outward currents between the preferred and orthogonal orientations, resembling the effect of TTX (Figures 4D, 4G, and 4H). For elongated bar stimuli, NBQX also eliminated the orientation-dependent difference in spiking responses, and the corresponding OBI analysis confirmed a marked reduction in spike-based orientation tuning (Figures 4E and 4F).

**Figure 4:**
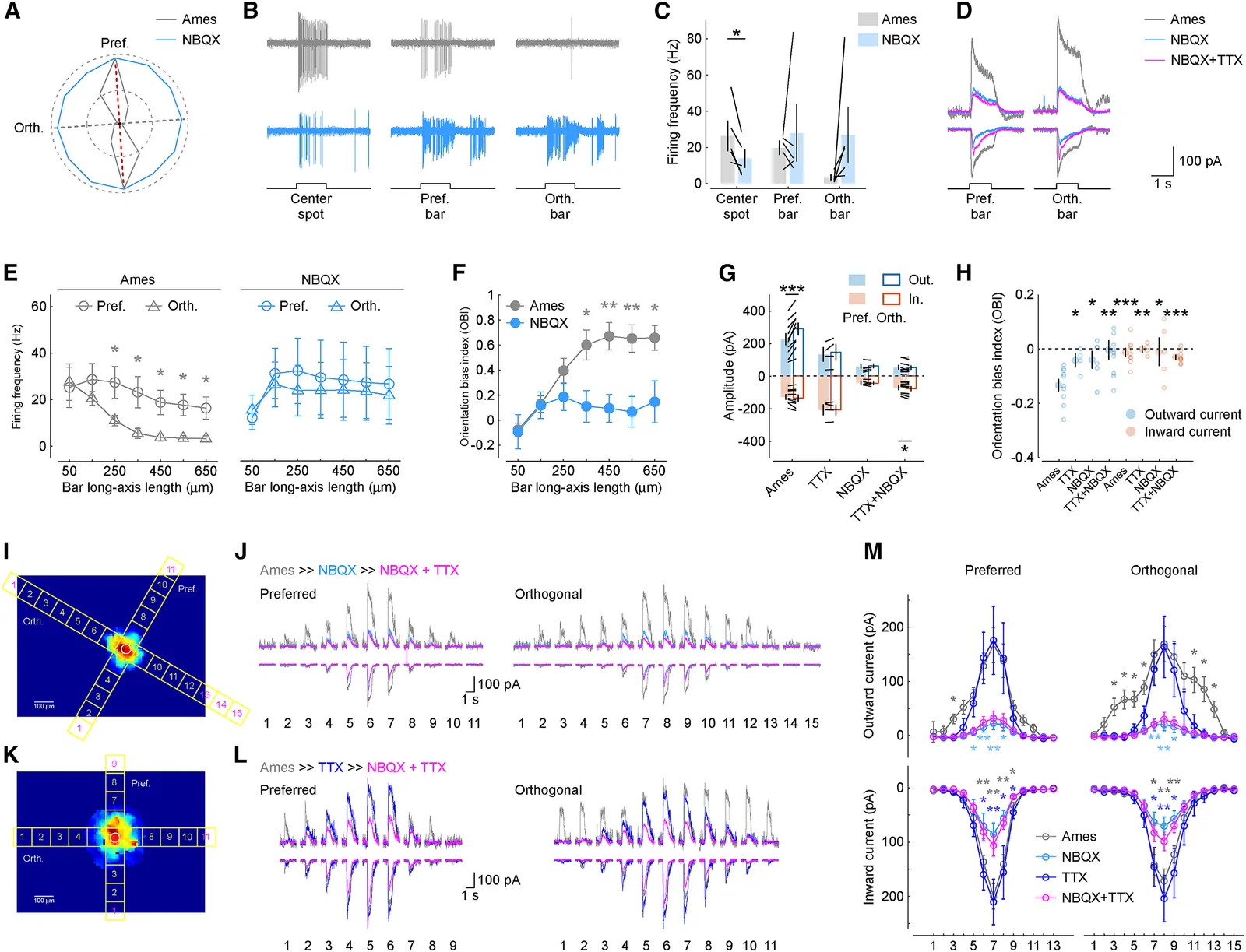
TTX-sensitive surround inhibition in S345 OSGCs requires AMPA/kainate receptor activation (A) Polar plots of spike responses from an S345 OSGC to oriented bar stimuli under control (Ames) and NBQX (5 μM) conditions. (B) Representative spiking activity of an S345 OSGC evoked by a light square (100 × 100 μm) and oriented bars (50 × 500 μm) along the preferred and orthogonal axes under control (Ames) and NBQX. (C) Comparison of OSGC firing rate responses to a light square (100 × 100 μm) and oriented bars (50 × 500 μm) along the preferred and orthogonal axes under control (Ames) and NBQX. *n* = 5 cells from 5 mice; *p* = 0.0279; two-sided paired *t* test. (D) Representative whole-cell voltage-clamp recordings of inward and outward currents evoked by bars presented along the preferred and orthogonal orientations in an S345 OSGC under control (Ames), NBQX (5 μM), and NBQX (5 μM) + TTX (100 nM) conditions. (E) Averaged spiking responses of S345 OSGCs to elongated bar stimuli presented along the preferred and orthogonal orientations under control (Ames; left) and NBQX (right) conditions (*n* = 5 cells in each condition, from 5 mice). Under the Ames condition, *p* = 0.0480, 0.0408, 0.0304, 0.0326, and 0.0421; two-sided paired *t* test. (F) Orientation bias index (OBI) of S345 OSGCs in response to bar stimuli of different lengths under control (Ames) and NBQX conditions (*n* = 5 cells in each condition, from 5 mice). *p* = 0.0179, 0.0044, 0.0054, and 0.0216; two-sided paired *t* test. (G) Amplitudes of outward and inward currents evoked by bars presented along the preferred and orthogonal orientations under control (Ames), TTX (100 nM), NBQX, and TTX + NBQX conditions (*n* = 10, 4, 6, and 10 cells, respectively, from 10 mice). *p* = 3.8 × 10^−5^ (Ames, outward), and 0.0157 (TTX + NBQX, inward); two-sided paired *t* test. (H) OBI of mean inward and outward currents evoked by bars presented along the preferred and orthogonal orientations under the same four pharmacological conditions in G (*n* = 10, 4, 6, and 10 cells, respectively, from 10 mice). OBI values derived from outward currents in Ames were compared with those from other conditions: *p* = 0.0257, 0.0177, 0.0033, 0.0002, 0.0022, 0.0137, and 0.0003; two-sided unpaired *t* test. (I and J) Excitatory receptive field of an OSGC and mapping stimuli along preferred and orthogonal axes (I), and corresponding currents evoked by focal stimulation at positions offset from the receptive field center under control (Ames), NBQX (5 μM), and NBQX (5 μM) + TTX (100 nM) (J). Scale bars, 100 μm. (K and L) Same as in (I) and (J) but recorded under control (Ames), TTX (100 nM), and NBQX (5 μM) + TTX (100 nM). Scale bars, 100 μm. (M) Population summary of currents evoked by local stimulation at different distances from the receptive field center along the preferred and orthogonal axes under different pharmacological conditions. For the Ames, NBQX (5 μM), TTX (100 nM), and NBQX + TTX conditions, *n* = 10, 6, 4, and 10 cells, respectively, from 10 mice. For outward currents: along the preferred axis, gray asterisks denote Ames vs. TTX (*p* = 0.0275); cyan asterisks denote NBQX vs. TTX (*p* = 0.0191, 0.0054, 0.0095, 0.0304). Along the orthogonal axis, gray asterisks denote Ames vs. TTX (*p* = 0.0479, 0.0230, 0.0279, 0.0355, 0.0408, 0.0375, 0.0494); cyan asterisks denote NBQX vs. TTX (*p* = 0.0028, 0.0080, 0.0193). For inward currents: along the preferred axis, gray asterisks denote Ames vs. NBQX (*p* = 0.0098, 0.0032, 0.0032, 0.0248); blue asterisks denote TTX vs. NBQX (*p* = 0.0282, 0.0097, 0.0282, 0.0109). Along the orthogonal axis, gray asterisks denote Ames vs. NBQX (*p* = 0.0105, 0.0039, 0.0095); blue asterisks denote TTX vs. NBQX (*p* = 0.0340, 0.0049, 0.0215). Two-sided unpaired *t* test. ∗*p* < 0.05, ∗∗*p* < 0.01, ∗∗∗*p* < 0.001. Data are shown as mean ± SEM.

We next dissected the central versus surround contributions to the OSGC RF (Figures 4I and 4K). Applying NBQX first markedly reduced both excitation and inhibition in the RF center along both axes and nearly abolished distal inhibition; subsequent TTX produced no further change in the remaining central excitation or inhibition (Figures 4J, 4M, S8A, and S8B). Reversing the order—TTX first—selectively reduced or eliminated distal inhibition along both axes, with little effect on central excitation or inhibition; adding NBQX thereafter caused a pronounced reduction of the central excitatory and inhibitory inputs (Figures 4L, 4M, S8C, and S8D). These sequence-dependent effects indicate distinct sources for central versus surround inhibition.

Together, these findings support the following picture: ON-sustained OSGCs possess a symmetric excitatory RF center that is AMPA/kainate dependent and accompanied by local inhibition that is likewise regulated by AMPA/kainate signaling. By contrast, OS is shaped by an asymmetric inhibitory surround that is spatially elongated along the orthogonal axis, GABAergic, and action-potential dependent, and whose recruitment involves AMPA/kainate-driven circuits upstream. Thus, blocking AMPA/kainate signaling disrupts the surround component and reduces OS, while leaving a residual inward current whose source remains to be determined.

### Spiking OSACs show asymmetric inward current responses

We next examined whether spiking amacrine cells in the mouse retina can carry orientation-selective signals and may participate in ON orientation-selective retinal circuits. During our recordings, we identified physiologically defined OSACs with sustained ON spiking and orientation-tuned responses (Figure 5A).

**Figure 5:**
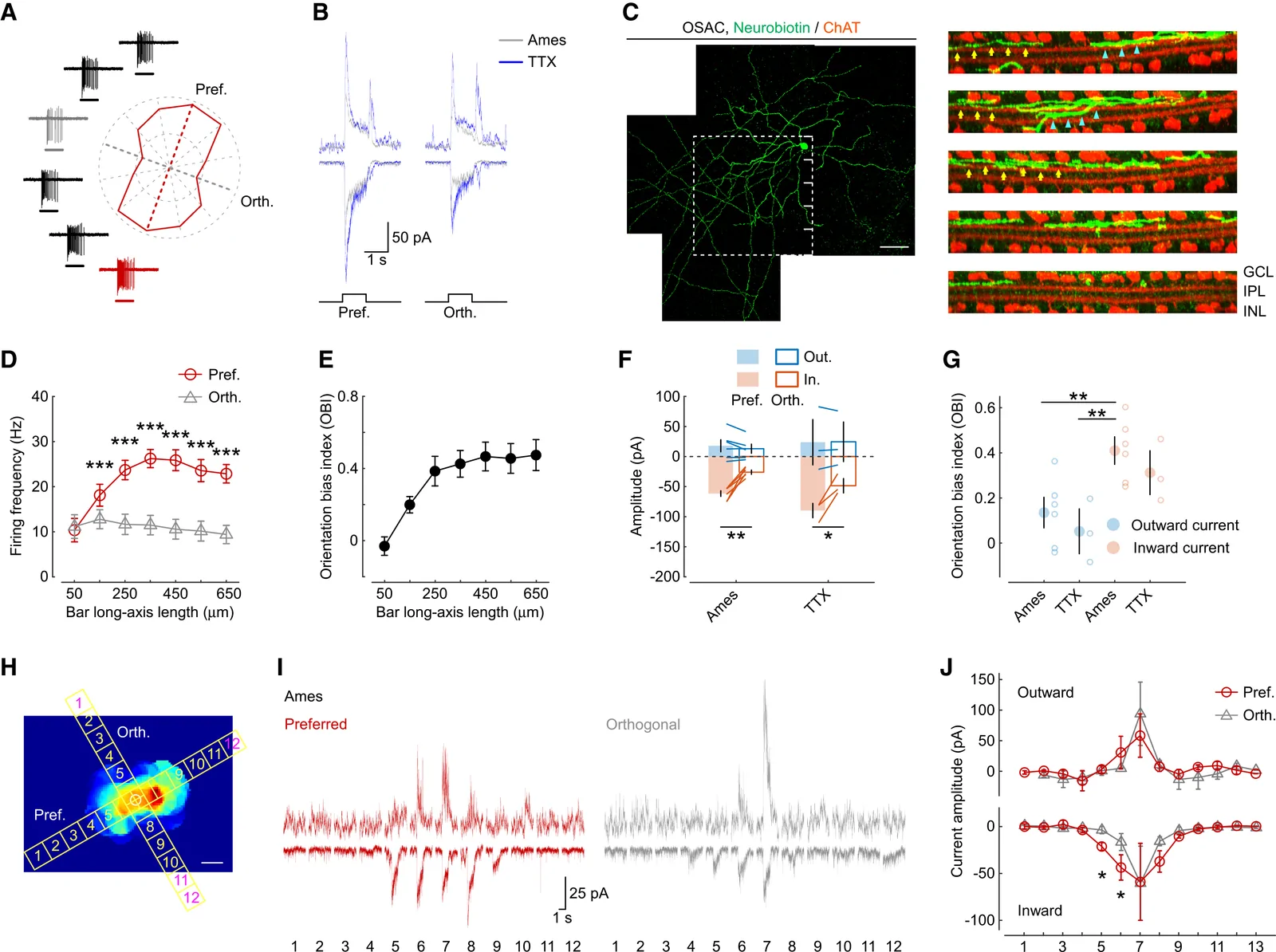
OSACs exhibit elongated excitatory receptive fields along the preferred orientation (A) Polar plots showing spiking responses of an OSAC to bar stimuli presented at different orientations. Red, preferred orientation; gray, orthogonal orientation. (B) Representative inward and outward currents recorded from an OSAC in response to bar stimuli presented along the preferred and orthogonal orientations under control (Ames) and TTX (100 nM) conditions. (C) Morphology of a representative OSAC (left, green) and its dendritic stratification within the inner plexiform layer (right, green). ChAT immunostaining (red) delineates the ON and OFF sublaminae. Yellow arrows indicate axons of the OSAC, and cyan arrowheads mark its dendrites. Scale bars, 50 μm. (D) Averaged spiking responses of OSACs to elongated bar stimuli, comparing responses along preferred and orthogonal orientations. *n* = 8 cells from 8 mice, *p* = 0.0005, 0.00004, 0.00004, 0.00002, 0.00003 and 0.00001; two-sided paired *t* test. (E) Orientation bias index (OBI) of OSACs in response to bar stimuli of varying lengths. *n* = 8 cells from 8 mice. (F) Amplitudes of outward and inward currents evoked by bars presented along the preferred and orthogonal orientations under control (Ames) and TTX (100 nM). For the Ames and TTX conditions, *n* = 6 and 3 cells, respectively, from 6 mice; *p* = 0.0011 (Ames, inward currents) and 0.0341 (TTX, inward currents), two-sided paired *t* test. (G) OBI of mean inward and outward currents evoked by bars presented along the preferred and orthogonal orientations under the control (Ames) and TTX conditions. For the Ames and TTX conditions, *n* = 6 and 3 cells, respectively, from 6 mice. OBI values derived from inward currents in Ames were compared with those from other conditions: *p* = 0.0079 and 0.0078; two-sided unpaired *t* test. (H and I) Excitatory receptive field of an OSAC and mapping stimuli along preferred and orthogonal axes (H), and corresponding currents evoked by focal stimulation at positions offset from the receptive field center under control (Ames) (I). Scale bars, 100 μm. (J) Comparison of average inward and outward current amplitudes evoked by local light stimulation (100 × 100 μm) at different distances from the receptive field center along the preferred and orthogonal axes. *n* = 3 cells from 3 mice, *p* = 0.0484 and 0.0170 (inward currents, pref. vs. orth.); two-sided paired *t* test. ∗*p* < 0.05, ∗∗*p* < 0.01, ∗∗∗*p* < 0.001. Data are shown as mean ± SEM.

Compared with orthogonal bars, stimuli aligned with the preferred orientation evoked larger inward currents, whereas outward currents showed little orientation dependence (Figure 5B). In the presence of TTX, the orientation bias of inward currents persisted (Figures 5F and 5G), suggesting that this orientation-tuned inward current component does not require TTX-sensitive spiking activity in upstream retinal circuits. Morphologically, recorded OSACs had sparse but wide dendritic fields and extensive, multidirectional axons (Figure 5C, left). Dendrites spanned IPL strata S1, S3, and S5 (Figure 5C, right, cyan arrowheads), whereas axons were mainly confined to S5 (Figure 5C, right, yellow arrows). Because their axonal arbors were mainly confined to S5, these cells may be anatomically positioned to participate in ON-layer orientation-selective retinal circuits, although their direct postsynaptic targets were not determined.

Spiking responses increased with bar length along the preferred axis, rising monotonically and saturating at approximately 250 μm, while remaining nearly constant along the orthogonal axis (Figure 5D). Orientation differences emerged at 150 μm, and the OBI exceeded 0.3 at 250 μm (Figure 5E). Consistent with the spiking responses, inward currents showed a stronger orientation bias than outward currents under both control and TTX conditions (Figures 5F and 5G), indicating that orientation tuning in OSACs was associated with asymmetric inward current responses.

RF mapping further showed that inward currents extended farther and had larger amplitudes along the preferred axis (Figures 5H–5J), whereas outward currents were more compact, centered near the soma, and less orientation dependent (Figures 5I and 5J).

Together, these results show that physiologically identified spiking OSACs exhibit orientation-tuned spiking responses and asymmetric inward current responses. In limited recordings, NBQX reduced OSAC light-evoked responses (Figure S9), suggesting that AMPA/kainate receptor-mediated signaling contributes to their light responses. However, the effect of AMPA/kainate receptor blockade on OSAC OBI was not quantified at the population level.

## Discussion

Our findings support the view that ON retinal OS can be implemented through more than one cellular and circuit mechanism. We characterized S345 OSGCs together with two less frequently recorded groups, putative S13 and S15 OSGCs, which differed in dendritic field size, IPL stratification, and responses to bars of different lengths. These observations suggest that ON orientation-selective signals are represented by multiple morphophysiologically distinguishable groups with different spatial integration properties, although the present data do not establish that they form independent parallel pathways. Mechanistic analyses focused on S345 OSGCs and revealed a prominent contribution of spike-dependent inhibition to their orientation tuning (Figure 6). We also recorded physiologically identified spiking OSACs with orientation-dependent inward-current responses, suggesting that orientation-selective processing may occur at multiple levels of the inner retinal circuit.

**Figure 6:**
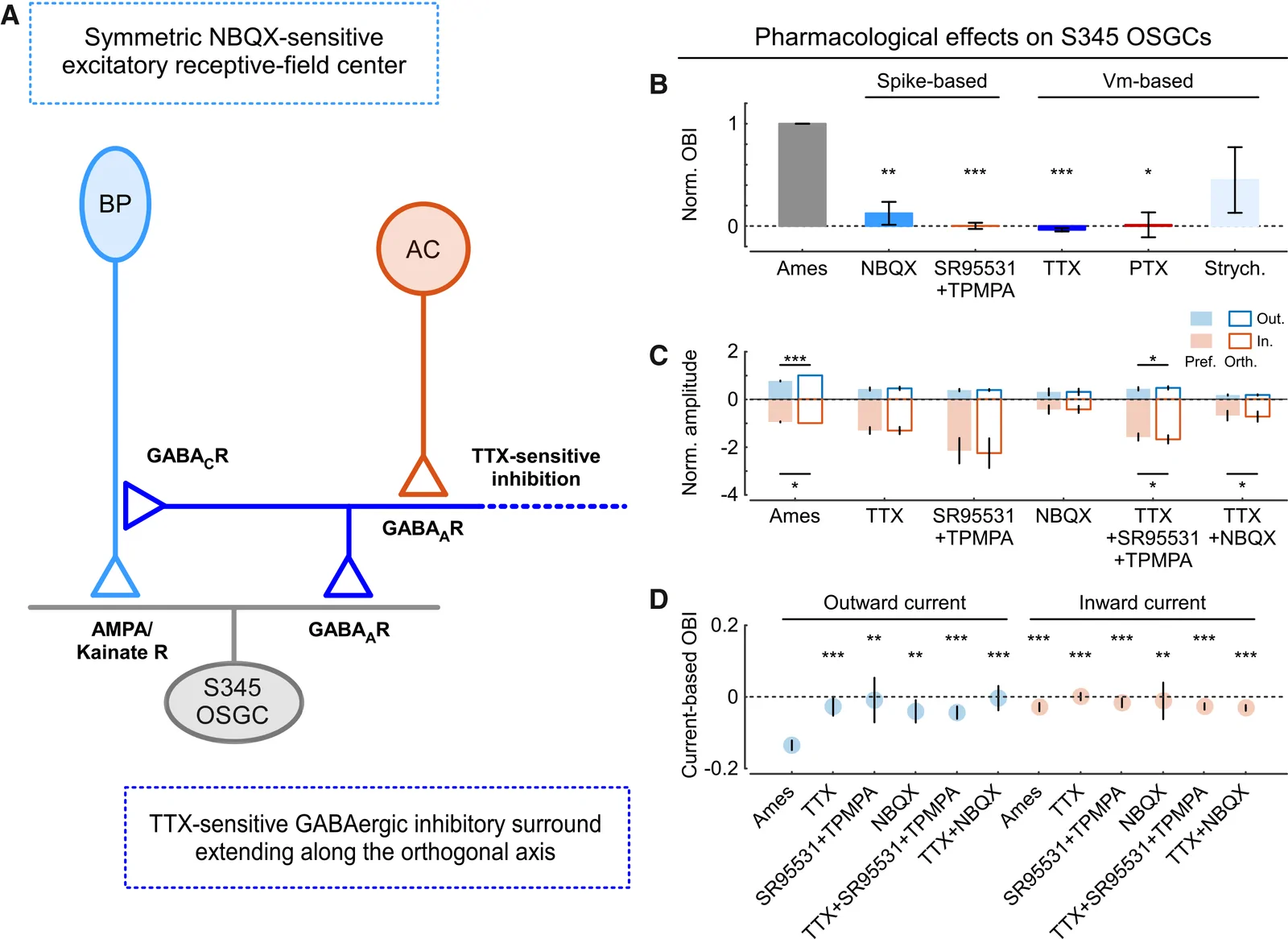
Proposed retinal circuitry and pharmacological modulation of orientation selectivity in S345 OSGCs (A) Proposed retinal circuitry supporting orientation selectivity in S345 OSGCs. AC, amacrine cell; BP, bipolar cell; OSGC, orientation-selective ganglion cell. (B) Summary of the effects of bath-applied NBQX (5 μM), SR95531 (5 μM) + TPMPA (50 μM), TTX (100 nM), picrotoxin (PTX; 100 μM), and strychnine (Strych.; 2 μM) on the OBI of S345 OSGCs. OBI was calculated from spiking responses for NBQX and SR95531 + TPMPA and from membrane potential responses for TTX, PTX, and Strych. For each cell, OBI after drug application was normalized to its corresponding control value in Ames’ medium, which was set to 1. *n* = 5 cells for NBQX, 5 for SR95531 + TPMPA, 5 for TTX, 3 for PTX, and 3 for Strych., obtained from a total of 21 mice. Compared with the corresponding control condition, *p* = 0.0014 for NBQX, 5.1 × 10^−6^ for SR95531 + TPMPA, 3.6 × 10^−7^ for TTX, 0.0148 for PTX, and 0.2286 for Strych.; two-sided paired t-tests. The TTX, PTX, and Strych. datasets are the same as those shown in Figures 2E, S7C, and S7F, respectively. (C) Summary of the effects of different pharmacological treatments on the outward and inward currents evoked by bars presented along the preferred and orthogonal orientations. For each cell, outward currents were normalized to the outward current evoked by an orthogonal-orientation bar under the control Ames condition, which was set to 1, whereas inward currents were divided by the absolute value of the inward current evoked by an orthogonally oriented bar under control (Ames) conditions, setting that response to −1. *n* = 19 cells for Ames, 9 for TTX, 4 for SR95531 + TPMPA, 6 for NBQX, 9 for TTX + SR95531 + TPMPA, and 10 for TTX + NBQX, obtained from a total of 19 mice. Comparisons between preferred- and orthogonal-orientation responses yielded *p* = 7.1 × 10^−10^ (Ames, outward), *p* = 0.0428 (TTX + SR95531 + TPMPA, outward), *p* = 0.0272 (Ames, inward), *p* = 0.0117 (TTX + SR95531 + TPMPA, inward), and *p* = 0.0219 (TTX + NBQX, inward); two-sided paired *t* test. The datasets summarized here are the same as those presented in Figures 3F and 4G. (D) Summary of the orientation bias index (OBI) calculated separately for each cell from the normalized inward and outward currents shown in (C) under the same pharmacological conditions (*n* = 19, 9, 4, 6, 9, and 10 cells, respectively, from 19 mice). The OBI calculated from normalized outward currents under the Ames condition was compared with the OBI values calculated from normalized inward and outward currents under the other conditions; *p* = 0.0002, 0.0024, 0.0027, 0.0003, 0.0001, 4.0 × 10^−7^, 3.9 × 10^−7^, 0.0006, 0.0014, 1.1 × 10^−5^, and 1.6 × 10^−5^; two-sided unpaired *t* test. ∗*p* < 0.05, ∗∗*p* < 0.01, ∗∗∗*p* < 0.001. Data are shown as mean ± SEM.

A key feature of S345 OSGCs is their dependence on spatially asymmetric inhibition. These cells are stratified continuously within IPL strata S3–S5, including S4, but show little direction selectivity under moving stimuli. Thus, although their dendrites overlap the choline acetyltransferase (ChAT) bands, their responses do not support a simple assignment to the canonical SAC-mediated direction-selective circuitry.^29^ Instead, our physiological and pharmacological results support a RF organization comprising a relatively compact excitatory center and an inhibitory surround extending farther along the orthogonal axis. TTX abolished orientation tuning and selectively suppressed distal outward-current responses, while GABA receptor blockade strongly reduced OS and attenuated the orientation-dependent difference in outward currents. By comparison, strychnine produced only a modest effect. These results indicate that action potential-dependent GABAergic inhibition is a key contributor to OS in S345 OSGCs. NBQX also reduced the orientation-dependent inhibitory component and eliminated spike-based tuning, consistent with AMPA/kainate receptor-dependent recruitment of the inhibitory circuit. The secondary spike burst observed under NBQX may reflect partial disinhibition resulting from reduced recruitment of inhibitory amacrine-cell circuitry, thereby unmasking a delayed component of residual excitatory drive.^30^^,^^31^ However, because NBQX affected both inward and outward currents and this secondary response component was not pharmacologically isolated, its precise circuit origin, as well as the interneurons and synaptic sites responsible for the effects of NBQX, remains uncertain.

S345 OSGCs share sustained ON responses and cardinal orientation preferences with previously described mouse ON OSGCs but differ in several respects. In the previously reported cells, preferred-orientation excitation was prominent; horizontal-preferring cells also showed orthogonally tuned inhibition and elongated dendritic fields associated with their preferred orientation, whereas vertical-preferring cells were more symmetric.^19^ In S345 OSGCs, horizontal-preferring cells were instead more symmetric, while vertical-preferring cells showed greater dendritic elongation; however, spike-based OBI did not differ significantly between groups and was not significantly correlated with morphology-based OBI. Although the two subgroups showed comparable spike-based OBI and broadly similar length-dependent response profiles, the available mechanistic datasets were insufficient to determine whether horizontal- and vertical-preferring S345 OSGCs rely on identical synaptic mechanisms. Moreover, TTX did not eliminate orientation-selective inhibition in the previously described ON OSGCs, whereas S345 tuning depended strongly on a TTX-sensitive GABAergic surround. Together with their continuous S3–S5 stratification, these differences support the identification of S345 OSGCs as a morphophysiologically distinguishable ON-OSGC group.

The less frequently recorded putative S13 and S15 groups further illustrate the diversity of mouse ON OSGCs. Putative S13 OSGCs had relatively compact dendritic fields, stratified mainly in S1 and S3, and exhibited comparatively transient ON responses. S15 OSGCs had broader dendritic fields, stratified in S1 and the inner IPL near S4/S5, and showed sustained ON responses with an additional offset component. Their bistratified morphology and response properties partially resemble previously reported mouse ON OSGCs.^19^ However, because both groups were encountered infrequently, larger datasets and molecular characterization will be required to establish their cell-type identities and relationships to previously characterized RGC populations.

Across species and RGC classes, S345 OSGCs share with rabbit OSGCs a dependence on GABAergic presynaptic circuitry, consistent with earlier evidence that retinal OS can involve lateral inhibition, dendritic asymmetry, and spatially organized excitatory and inhibitory subfields.^11^^,^^12^^,^^13^^,^^14^^,^^15^^,^^26^ However, S345 OSGCs differ in their stratification, RF organization, and strong dependence on TTX-sensitive surround inhibition. Mouse OFF OSGCs provide a further contrast, as their OS can be conveyed through electrical coupling with asymmetric amacrine cells rather than the GABAergic inhibitory mechanism implicated here.^18^ These comparisons highlight the diversity of retinal mechanisms supporting OS.

The spiking OSACs recorded here showed sustained ON responses and stronger inward-current responses to preferred than orthogonal bars, with this orientation bias persisting in TTX. RF mapping under control conditions further showed that inward currents extended farther along the preferred axis. These findings associate their orientation-tuned spiking with asymmetric inward-current responses but do not establish that such currents generate OS. However, because the RF current comparison in Figure 5J was based on only three cells, this result should be considered preliminary. In addition, the available NBQX recordings were insufficient for a population-level analysis of OSAC OBI. Thus, although limited recordings indicated that NBQX reduced OSAC light-evoked responses, whether AMPA/kainate receptor-mediated currents are required for OSAC OS remains unresolved. Their broad dendritic fields and S5-enriched axons resemble some features of rabbit wide-field and polyaxonal OSACs,^14^^,^^16^ but their precise cell-type identity and postsynaptic targets remain unresolved. Although their axonal stratification suggests possible participation in ON-layer OS circuitry, no direct connection to S345 OSGCs was demonstrated. Because our recordings were obtained mainly from a restricted region of the temporal hemiretina and the precise retinal coordinates of individual cells were not systematically documented, the observed proportions and orientation preferences of OSGCs and OSACs should not be generalized to the entire retina. Their distribution within the global retinal orientation-selectivity map therefore remains unknown.^23^^,^^32^

In summary, our results identify S345 OSGCs as a morphophysiologically identifiable ON orientation-selective group in which TTX-sensitive GABAergic surround inhibition strongly shapes orientation tuning. The recorded spiking OSACs reveal an additional pattern of orientation-selective processing associated with asymmetric inward-current responses, although their cell-type identity, causal mechanism, and relationship to S345 OSGCs remain unresolved. These findings expand the known range of cellular strategies associated with retinal OS and provide a basis for identifying the specific interneurons and connections underlying OS in S345 OSGCs.

### Limitations of the study

Small samples of putative S13 OSGCs, S15 OSGCs, and spiking OSACs limited systematic comparisons across cell groups. The lack of established molecular markers necessitated physiological screening, limiting cell yield and opportunities for paired recordings between OSACs and S345 OSGCs. Their direct connectivity remains unverified, and the specific interneurons and synaptic sites underlying the pharmacological effects remain unresolved. Finally, only male mice were studied; potential sex differences were not assessed, and the applicability of these findings to females remains to be established.

## Resource availability

### Lead contact

Requests for further information and resources should be directed to and will be fulfilled by the lead contact, Le Sun (sunle@ccmu.edu.cn).

### Materials availability

This study did not generate new, unique reagents.

### Data and code availability

• Data supporting the findings of this study are available within the article and supplemental information. Additional underlying data may be obtained from the lead contact upon reasonable request. Requests will be considered based on their scientific purpose, scope, and technical feasibility.
• This study did not generate standardized datasets requiring deposition in a public repository.
• The analysis code is publicly available on GitHub (https://github.com/SunLab-code/OSGCs) and archived on Zenodo (https://doi.org/10.5281/zenodo.23430091).
• Any additional information required to reanalyze the data reported in this paper is available from the lead contact upon reasonable request.

## Acknowledgments

This work was supported by the National Natural Science Foundation of China (NSFC) (32171021).

## Author contributions

S.H. and L.S. conceived and supervised the project. X.H., S.H., and L.S. designed the experiments and discussed the results. X.H. and L.S. performed the experiments and the analysis. X.H., S.H., and L.S. wrote the manuscript, and all authors edited and proofread the manuscript.

## Declaration of interests

The authors declare no competing interests.

## Declaration of generative AI and AI-assisted technologies in the manuscript preparation process

During the preparation of this work, the authors used ChatGPT (OpenAI) to assist with manuscript translation and language editing. After using this tool, the authors reviewed and edited the content as needed and take full responsibility for the content of the published article.

## STAR★Methods

### Key resources table

**Table:** 

REAGENT or RESOURCE	SOURCE	IDENTIFIER
**Antibodies**		

goat anti-choline acetyltransferase (ChAT)	Chemicon	Cat# AB144P; 1:200; RRID: AB_2079751
Streptavidin, Fluorescein	Vector Laboratories	Cat# SA-5001–1; 1:200

**Chemicals, peptides, and recombinant proteins**		

Ames	Sigma-Aldrich	Cat# A1420
Neurobiotin	Vector Laboratories	Cat# SP-1120; 0.5%
Lucifer Yellow	Sigma-Aldrich	Cat# L0259; 0.1%
Picrotoxin	Tocris Bioscience	Cat# 1128; 100 μM
SR95531	Sigma-Aldrich	Cat# S106; 5 μM
TPMPA	Sigma-Aldrich	Cat# T200; 50 μM
Strychnine	Sigma-Aldrich	Cat# S8753; 2 μM
TTX	Sigma-Aldrich	Cat# 554412; 100 nM
NBQX	Sigma-Aldrich	Cat# N183; 5 μM

**Critical commercial assays**		

FluoView FV500 confocal microscope	Olympus	Model FV500
Axopatch 200B amplifier	Molecular Devices	Model Axopatch 200B; RRID: SCR_018866
Digidata 1322A digitizer	Molecular Devices	Model Digidata 1322A; RRID: SCR_021041

**Software and algorithms**		

ImageJ	NIH	RRID: SCR_003070
MATLAB	MathWorks	RRID:SCR_001622
pClamp 8.2	Molecular Devices	RRID: SCR_011323

**Deposited data**		

Custom analysis code for OSGCs	This paper	Zenodo: https://doi.org/10.5281/zenodo.23430091; GitHub: https://github.com/SunLab-code/OSGCs

### Experimental model and study participant details

#### Mice

Male wild-type C57BL/6J mice (4–8 weeks old) were used for all experiments. Animals were purchased from SPF (Beijing) Biotechnology Co., Ltd. (Beijing, China). According to the supplier’s health records, the mice had been maintained under specific pathogen-free (SPF) conditions. No overt signs of illness were observed during routine inspection upon arrival. Animals were housed under SPF conditions at 22 ± 2°C on a 12-h light/12-h dark cycle, with food and water available *ad libitum*. All animal procedures were approved by the Animal Experiments and Experimental Animal Welfare Committee of Capital Medical University (approval no. AEEI-2026–154) and conducted in accordance with the Guide for the Care and Use of Laboratory Animals.

### Method details

#### Electrophysiology

For electrophysiological recordings, mice were dark-adapted for at least 2 h prior to experiments. Animals were anesthetized with isoflurane and sacrificed by rapid cervical dislocation. Eyes were enucleated immediately, and a small incision was made at the temporal edge to preserve retinal orientation.^33^^,^^34^ Recordings were performed mainly in the temporal hemiretina, near the midpoint between the retinal center and the temporal retinal edge. Retinas were isolated, mounted ganglion-cell side up on black Millipore filters, and transferred to a 0.5-mL recording chamber continuously superfused with oxygenated, bicarbonate-buffered Ames’ medium (5 mL/min; Sigma-Aldrich, St. Louis, MO, USA). The chamber was mounted on the fixed stage of an upright microscope (E600FN, Nikon) equipped with epifluorescence optics and a 40× water-immersion objective. During cell targeting and patching, retinal cells were visualized using infrared differential interference contrast optics.

For cell screening and identification, the inner limiting membrane overlying the target region was removed using a glass micropipette filled with Ames’ medium to expose somata in the ganglion cell layer. Cells with medium-sized somata, typically approximately 10–15 μm in diameter, were sequentially screened by loose-patch recording. ON responses were first identified using flashing spot stimuli, receptive-field centers were mapped with small spot stimuli, and orientation selectivity was then tested using oriented bar stimuli centered on the receptive field. Cells with an OBI >0.3 were classified as ON-OSGCs and further examined by whole-cell recording and morphological recovery using Lucifer Yellow and neurobiotin. For identified ON-OSGCs, additional electrophysiological recordings were obtained in response to elongated bars of varying lengths and flashing squares presented along the preferred or orthogonal orientation. Final classification was based on both electrophysiological response properties and dendritic morphology. Using this procedure, we recorded 51 ON OSGCs, including 39 S345 OSGCs, 6 putative S13 OSGCs, and 6 S15 OSGCs, as well as 8 orientation-selective amacrine cells. For mechanistic analyses of S345 OSGCs, cells with medium-sized somata, medium-sized receptive fields, and sustained ON responses to spot stimuli were preferentially selected for further recording. Thus, the recorded dataset represents a targeted sampling of physiologically identified orientation-selective cells rather than an unbiased survey of OSGC abundance across the mouse retina.

After physiological identification, whole-cell recordings were performed using recording pipettes with a resistance of 8–12 MΩ. Pipettes were filled with either voltage-clamp internal solution (in mM: 120 Cs-methanesulfonate, 0.5 CaCl_2_, 5 EGTA, 10 HEPES, 4 ATP, 0.5 GTP, and 5 QX-314; pH 7.2 adjusted with 1 M CsOH) or current-clamp internal solution (in mM: 120 K-gluconate, 5 NaCl, 10 KCl, 1 MgCl_2_, 1 EGTA, 10 HEPES, 2 ATP, 5 QX-314, and 0.5 GTP; pH 7.2 adjusted with 1 M KOH). Both solutions contained 0.5% neurobiotin (Vector Laboratories) and 0.1% Lucifer Yellow (Sigma-Aldrich) to enable post hoc morphological identification and reconstruction. After a gigaohm seal (>1 GΩ) was established, the whole-cell configuration was obtained by rupturing the membrane patch. Input resistance was not systematically measured across all recorded cells and was therefore not included in comparisons among OSGC groups. The liquid junction potential of 10 mV was always corrected.

Depending on the experimental design, cells were stimulated with different light protocols and pharmacologically manipulated with one or more receptor antagonists added to Ames medium at the following concentrations: Picrotoxin (100 μM; Tocris Bioscience), SR95531 (5 μM; Sigma-Aldrich), TPMPA (50 μM; Sigma-Aldrich), Strychnine (2 μM; Sigma-Aldrich), TTX (100 nM; Sigma-Aldrich), and NBQX (5 μM; Sigma-Aldrich). After whole-cell recordings, the fluorescent tracers allowed visualization of gross cell morphology in relation to stimulus position.

Signals were amplified using an Axopatch 200B amplifier, digitized through a Digidata 1322A interface, and acquired with pCLAMP 8.2 software in gap-free mode at a sampling rate of 5 kHz (Molecular Devices). Each recording contained one channel for the electrophysiological signal and a second channel carrying a synchronization signal that indicated the timing of visual-stimulus presentation. Responses to repeated presentations of the same stimulus were aligned using this synchronization signal and averaged for subsequent analysis. For voltage-clamp recordings, the same visual-stimulus protocol was presented in separate trials while cells were held at −65 mV or +20 mV. Recordings at −65 mV were used to measure predominantly inward currents, whereas recordings at +20 mV were used to measure predominantly outward currents. Because only two holding potentials were used and synaptic reversal potentials were not determined in individual cells, excitatory and inhibitory conductances were not estimated. For current-clamp recordings, cells were recorded in I = 0 mode without current injection.

#### Visual stimuli

Visual stimuli were generated using custom software written in C# with DirectX and displayed on a Sony E230 monitor (resolution: 1024 × 768 pixels; refresh rate: 60 Hz). The stimulus intensity was 0.35 × 10^11^ photons cm^−2^ s^-1^.^35^ The monitor image was relayed through a condenser lens and reflected by a 45° semi-transparent mirror positioned beneath the recording chamber. This optical arrangement permitted infrared visualization during cell targeting and patching while directing visible stimulus light toward the retina. The stimulus image was focused onto the photoreceptor layer of the isolated retina. The optical system was calibrated such that one pixel on the display corresponded to approximately 1 × 1 μm on the retinal surface. Monitor contrast and color temperature were not independently measured. Visual stimulation consisted of five protocols.

##### Flashing spot stimulus

A 50-μm diameter spot was presented for 1 s to measure ON, OFF, and sustained versus transient light responses. Receptive field maps were constructed by recording cellular responses to different spot locations, allowing identification of the receptive field center.

##### Oriented bar stimulus

A 500 × 50 μm rectangular bar was presented for 1 s with 5-s intervals, centered on the receptive field. Bars were shown at six orientations, incremented in steps of 30°. Orientation selectivity was determined by comparing spiking responses across orientations, and the preferred and orthogonal orientations were identified.

##### Elongated bar of varying length stimulus

Rectangular bars with a fixed short edge of 50 μm and variable long edges (50–650 μm in 100-μm increments) were presented for 1 s with 5-s intervals, centered on the receptive field and extended along one of the six tested orientations. This stimulus was used to examine responses to stimuli of different spatial extents along a given orientation.

##### Moving square stimulus

A 100 × 100 μm square was swept across the receptive field center at 19 deg/s in 12 directions (30° increments), over a path length of 1500 μm. This stimulus was used to assess direction selectivity.

##### Flashing square along preferred or orthogonal orientation stimulus

A 100 × 100 μm square was flashed at 100-μm intervals along the preferred or orthogonal axis, including the receptive-field center. Stimulus duration was 1 s or 0.5 s, with corresponding inter-stimulus intervals of 4 s or 2 s. This stimulus was used to probe the influence of distal inputs on light responses.

#### Immunohistochemistry

Retinal preparations following electrophysiological recordings were immediately transferred into 4% paraformaldehyde and fixed at room temperature for 1 h. Tissues were rinsed three times (10 min each) in 0.01 M PBS, and then incubated with primary antibody (goat anti-ChAT, Chemicon, 1:200) diluted in antibody solution (per 20 mL: 200 mg BSA, 100 μL Triton X-100, 2 mg sodium azide in 0.01 M PBS) for 3 days at 4°C in the dark. After washing three times (10 min each) in 0.01 M PBS, tissues were incubated overnight at 4°C in the dark with secondary antibody (TRITC-conjugated anti-goat, 1:200) and fluorescein-conjugated streptavidin (Vector Laboratories), diluted in antibody solution (per 20 mL: 100 μL Triton X-100 and 2 mg sodium azide in 0.01 M PBS). Samples were rinsed three times (10 min each) in 0.01 M PBS, mounted, and imaged using a confocal microscope (Olympus FluoView FV500) to collect morphological data.

#### Data analysis

Electrophysiological data were analyzed using custom routines written in MATLAB (MathWorks). Automatic reading of pClamp data files was performed using the abfload module developed by Harald Hentschke (available from www.mathworks.com). Based on different stimulus paradigms, dedicated analysis scripts were developed. For loose-patch recordings, response strength was quantified as the mean firing rate (Hz), calculated by dividing the number of spikes during the stimulus period by the stimulus duration in seconds. For voltage- and current-clamp recordings, baseline was defined as the mean current or membrane potential, respectively, during the 1 s immediately preceding stimulus onset. Voltage-clamp responses were quantified as the mean current during the stimulus period minus baseline (pA), with outward currents positive and inward currents negative. Current-clamp responses were quantified as the mean membrane potential during the stimulus period minus baseline (mV), with positive and negative values indicating depolarization and hyperpolarization, respectively. Unless otherwise specified, responses were averaged across 3–5 repeated stimulus presentations before calculating OBI, and representative response traces show trial averages. For the analysis of OBI stability across repeated stimulus presentations (Figure S2B), OBI was calculated separately for each repetition.

##### For flashing spot stimulus

Loose-patch recordings were used to measure spike responses to spot stimuli at different locations. Responses were averaged across trials and mapped to generate receptive field maps.

##### For oriented bar stimulus

Loose-patch, voltage-clamp, and current-clamp recordings were analyzed. Orientation selectivity was quantified using the orientation bias index (OBI)^29^:

OBI=(Pref.−Orth)/(Pref.+Orth.)

where *Pref.* and *Orth.* denote responses to the preferred and orthogonal orientations, respectively. Cells with a spike-based OBI >0.3 were considered orientation-selective. Polar plots of response strength across orientations were fitted with a von Mises distribution:

$$R\left(x\right)=R_{max}e^{\left[k\;\cos\;2\left(x-\mu \right)\right]}/e^{k}$$

where R(x) is the response strength at orientation x, *R*_*max*_ is the maximal response, *μ* is the fitted preferred orientation, and *k* is a concentration parameter. The preferred orientation (Pref.) was defined as the tested orientation closest to *μ*, and the orthogonal orientation (Orth.) as the orientation perpendicular to Pref.

For Figure 1I, OBI values were fitted against preferred orientation using a baseline plus two axial von Mises functions and multistart nonlinear least squares (0.01 ≤ k ≤ 1). Cells were assigned to the nearest fitted axis, accounting for 180° periodicity, and component widths were expressed as full widths at half maximum (FWHMs).

##### For elongated bar of varying length stimulus

Loose-patch and current-clamp responses were quantified at each bar length and orientation as described above, using mean firing rate (Hz) and mean change in membrane potential relative to baseline (mV), respectively. Responses were averaged across cells at each bar length, separately for the preferred and orthogonal orientations.

##### For moving square stimulus

Loose-patch recordings were used to assess motion responses. Direction selectivity was quantified using the direction selectivity index (DSI),^34^ and cells with DSI >0.3 were classified as direction-selective and excluded from subsequent analyses.

##### For flashing square along preferred or orthogonal orientation stimulus

Voltage-clamp responses were quantified at each stimulus position as baseline-subtracted mean inward and outward current amplitudes (pA), as described above. Responses were averaged across cells at each position, separately for each current type and axis.

##### Morphological analysis

Dendritic field boundaries were manually outlined in ImageJ, and the enclosed areas were measured. Custom MATLAB code was used for ellipse fitting to obtain major (a) and minor (b) axis lengths. Eccentricity and morphology-based OBI were calculated as $\sqrt{1-{\left(\frac{b}{a}\right)}^{2}}$ and (*a*-*b*)/(*a*+*b*), respectively. Dendritic stratification was expressed as fractional IPL depth, with total IPL thickness normalized to 1.

### Quantification and statistical analysis

Statistical analyses were performed using MATLAB R2024a (MathWorks). Data are presented as mean ± standard error of the mean (SEM), unless otherwise stated. Exact sample sizes, reported as numbers of cells and contributing mice, and the statistical tests used for individual comparisons are provided in the corresponding figure legends. Comparisons between two independent groups were performed using two-sided unpaired t-tests, whereas comparisons between paired measurements were performed using two-sided paired t-tests.

Linear relationships in Figures 1P, S1P, and S2A were analyzed using ordinary least-squares linear regression with an intercept. Ordinary coefficients of determination (R^2^) and *p* values from two-sided Pearson correlation tests are reported. Observations with missing or nonfinite values were excluded separately for each variable pair. Shaded bands, where shown, indicate pointwise 95% confidence intervals for the fitted mean responses.

For Figure S2B, changes in orientation bias index (OBI) across five stimulus repetitions were analyzed separately in horizontal-preferring and vertical-preferring S345 OSGCs using linear mixed-effects models fitted by restricted maximum likelihood (REML). Stimulus repetition was included as a categorical fixed effect and cell identity as a random intercept. The overall effect of stimulus repetition was assessed using F-tests with Satterthwaite approximation of the denominator degrees of freedom. All available observations were included without imputation, and cells with incomplete repetitions were retained in the mixed-effects analyses. Group means and SEM were calculated using the available observations at each repetition. Between-group comparisons at each repetition were performed using two-sided Welch’s t-tests, with *p* values unadjusted for multiple comparisons.

Statistical significance was defined as *p* < 0.05. Where asterisks are used, significance levels are indicated as follows: ∗*p* < 0.05, ∗∗*p* < 0.01, and ∗∗∗*p* < 0.001.
