# Supplementary material for: Distinct cellular mechanisms underlie ON orientation selectivity in the mouse retina
Source: iScience. 2026 Nov 6;29(11):118130. doi: 10.1016/j.isci.2026.118130 (PMC13761252; doi:10.1016/j.isci.2026.118130)
Supplement: Supplementary file 1 [file mmc1.pdf]

iScience, Volume *29*

Supplemental information

Distinct cellular mechanisms underlie ON orientation selectivity in the mouse retina

Xu Han, Shigang He, and Le Sun

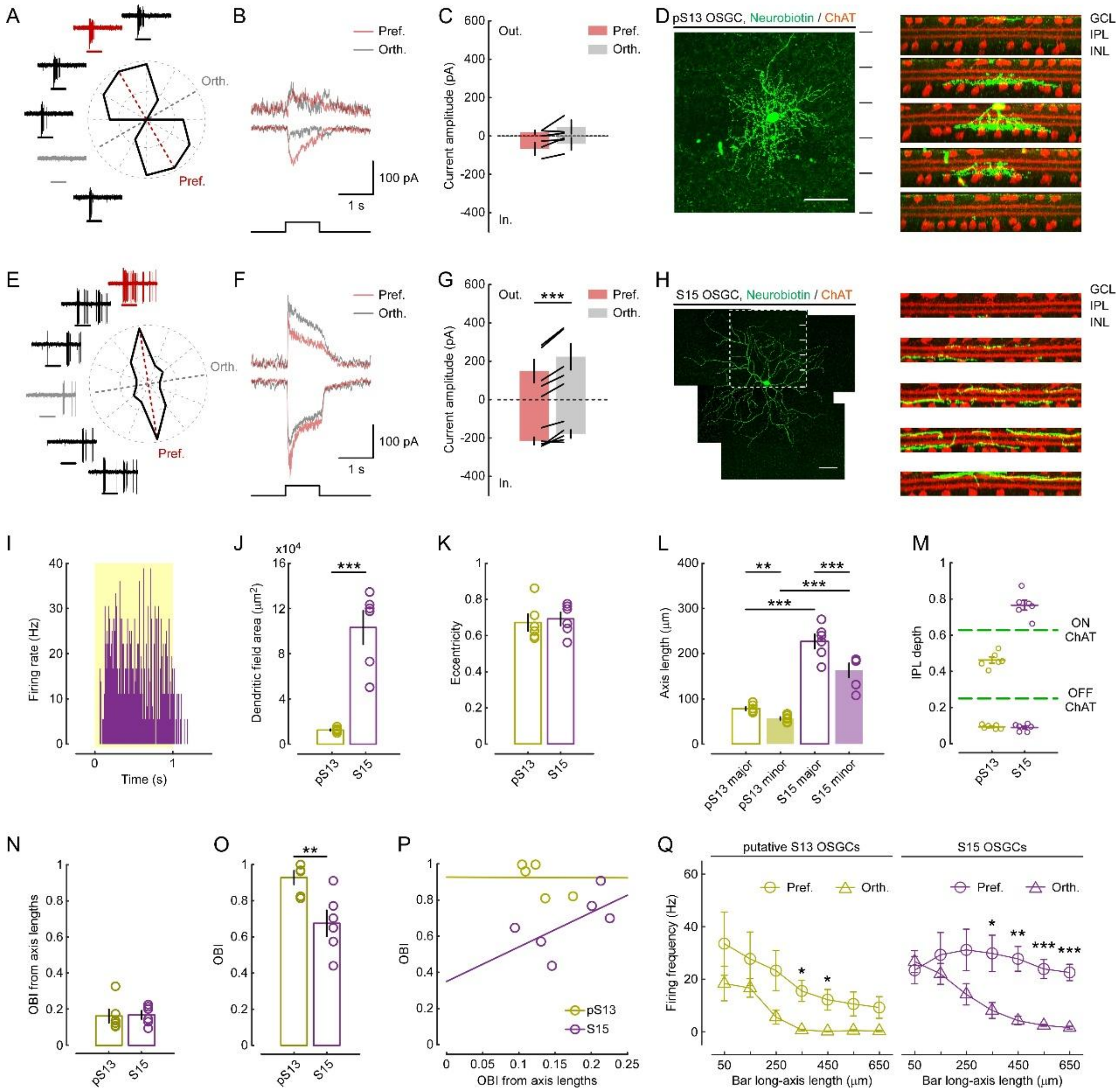

Figure S1: Physiological and Morphological Characterization of putative S13 (pS13) and S15 OSGCs. Related to figure 1.

(A, E) Polar plots showing spiking responses of ON-OSGCs to bar stimuli presented at different orientations. Representative spike trains are arranged along the corresponding stimulus orientations; horizontal lines below the traces indicate the 1-s stimulus duration. Red traces represent responses to bars moving along the preferred orientation, whereas gray traces represent responses to bars moving along the orthogonal orientation, which is perpendicular to the preferred axis. A, pS13 OSGCs; E, S15 OSGCs.

(B, F) Light-evoked inward and outward currents recorded from ON-OSGCs in response to bars presented along the preferred (red) and orthogonal (gray) orientations. Inward and outward currents were recorded under whole-cell voltage clamp at holding potentials of −65 mV and 20 mV, respectively. B, pS13 OSGCs; F, S15 OSGCs.

(C, G) Average amplitudes of light-evoked outward and inward currents in pS13 (C) and S15 (G) OSGCs in response to 1-s bar stimuli presented along the preferred (red) and orthogonal (gray) orientations. In C, n = 3 cells from 3 mice, p = 0.3664 and 0.1546 for outward and inward currents, respectively. In G, n = 5 cells from 5 mice, p = 0.00061 and 0.0693 for outward and inward currents, respectively. Two-sided paired t-test.

(D, H) Morphology of ON-OSGCs (left, green) and dendritic stratification within the inner plexiform layer (right, green). ChAT immunostaining (red) delineates ON and OFF bands. Scale bar, 50 µm.

(I) PSTH (10-ms bins) of spike responses in S15 OSGCs evoked by a 1-s light spot (50 µm in diameter) presented at the receptive-field center, averaged across 6 cells from 6 mice. The yellow-shaded area indicates the light-stimulation period.

(J) Comparison of dendritic field areas between pS13 and S15 OSGCs. For pS13 and S15 OSGCs, n = 6 cells from 6 mice in each group; $p = 5.8 \times 10^{-5}$. Two-sided unpaired t-test.

(K) Comparison of eccentricity after fitting the dendritic fields of pS13 and S15 OSGCs with ellipses. For pS13 and S15 OSGCs, n = 6 cells from 6 mice in each group; p = 0.7187. Two-sided unpaired t-test.

(L) Comparison of the major and minor axes of ellipse-fitted dendritic fields in pS13 and S15 OSGCs. For pS13 and S15 OSGCs, n = 6 cells from 6 mice in each group; p = 0.0064 for the major versus minor axes in pS13 OSGCs, p = 0.0003 for the major versus minor axes in S15 OSGCs; two-sided paired t-test. $p = 2.3 \times 10^{-6}$ for the major axes between pS13 and S15 OSGCs, and $p = 2.4 \times 10^{-5}$ for the minor axes between pS13 and S15 OSGCs; two-sided unpaired t-test.

(M) Distribution of the bistratified dendritic arbors of pS13 and S15 OSGCs within the IPL. For pS13 and S15 OSGCs, n = 6 cells from 6 mice in each group.

(N) OBI calculated from the major and minor axes of the dendritic fields. For pS13 and S15 OSGCs, n = 6 cells from 6 mice in each group; p = 0.8856. Two-sided unpaired t-test.

(O) OBI calculated from light-evoked spiking responses. For pS13 and S15 OSGCs, n = 6 cells from 6 mice in each group; p = 0.0070. Two-sided unpaired t-test.

(P) Linear correlation between OBI calculated from light-evoked spiking responses and OBI calculated from the major and minor axes of dendritic fields. For pS13 OSGCs, n = 6 cells from 6 mice, $R^2 = 0.00028$, p = 0.9748; for S15 OSGCs, n = 6 cells from 6 mice, $R^2 = 0.3811$, p = 0.1916.

(Q) Averaged spiking responses of ON-OSGCs to elongated bar stimuli presented along the preferred and orthogonal orientations. For pS13 OSGCs, n = 4 cells from 4 mice, p = 0.0106 and 0.0215; for S15 OSGCs, n = 5 cells from 5 mice, p = 0.0205, 0.0015, 0.00036, and 0.00016. Two-sided paired t-test.

*p < 0.05, **p < 0.01, ***p < 0.001. Data are shown as mean ± s.e.m.

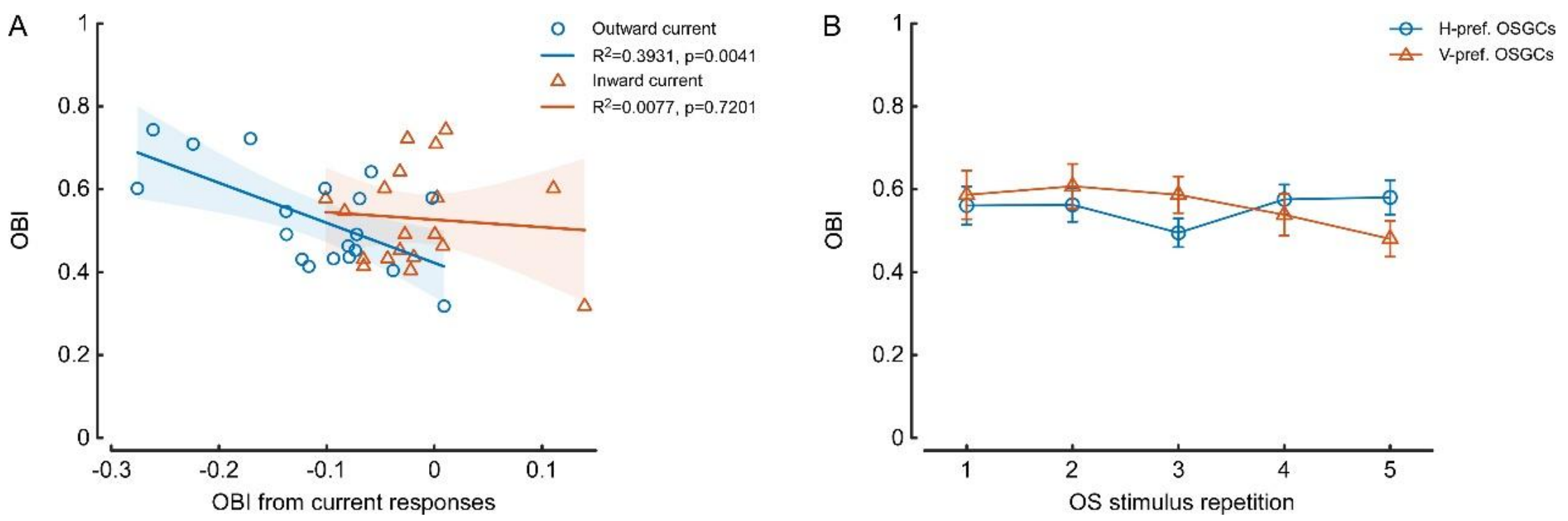

Figure S2: Relationships between current-based and spike-based OBI and stability of OBI across repeated oriented-bar stimuli in S345 OSGCs. Related to figure 1.

(A) Current-based OBI was calculated from inward or outward currents evoked by bars presented at the preferred and orthogonal orientations. Each symbol represents one cell (n = 19 cells from 19 mice). Blue open circles show outward-current OBI plotted against spike-based OBI, with the blue line indicating the linear fit ($R^2 = 0.3931$, p = 0.0041). Orange open triangles show inward-current OBI plotted against spike-based OBI, with the orange line indicating the linear fit ($R^2 = 0.0077$, p = 0.7201). p values were calculated using two-sided Pearson correlation tests.

(B) Orientation bias across repeated oriented-bar stimulus presentations in S345 OSGCs. Mean OBI values are shown for horizontal-preferring OSGCs (H-pref.; blue filled circles) and vertical-preferring OSGCs (V-pref.; orange filled triangles) across five stimulus repetitions. For repetitions 1–3, n = 29 cells from 29 mice for H-pref. and 10 cells from 10 mice for V-pref.; for repetitions 4–5, n = 27 cells from 27 mice and 9 cells from 9 mice, respectively. No significant effect of stimulus repetition was detected in either group using linear mixed-effects models with repetition as a categorical fixed effect and cell identity as a random intercept (H-pref.: $F(4, 108.1) = 1.945$, p = 0.1082; V-pref.: $F(4, 33.5) = 1.199$, p = 0.3293). No significant between-group differences were detected at any repetition (two-sided Welch's t-tests; unadjusted p = 0.7334, 0.5139, 0.1170, 0.5564, and 0.1073 for repetitions 1–5, respectively). Data are presented as mean ± SEM; lines connect group means.

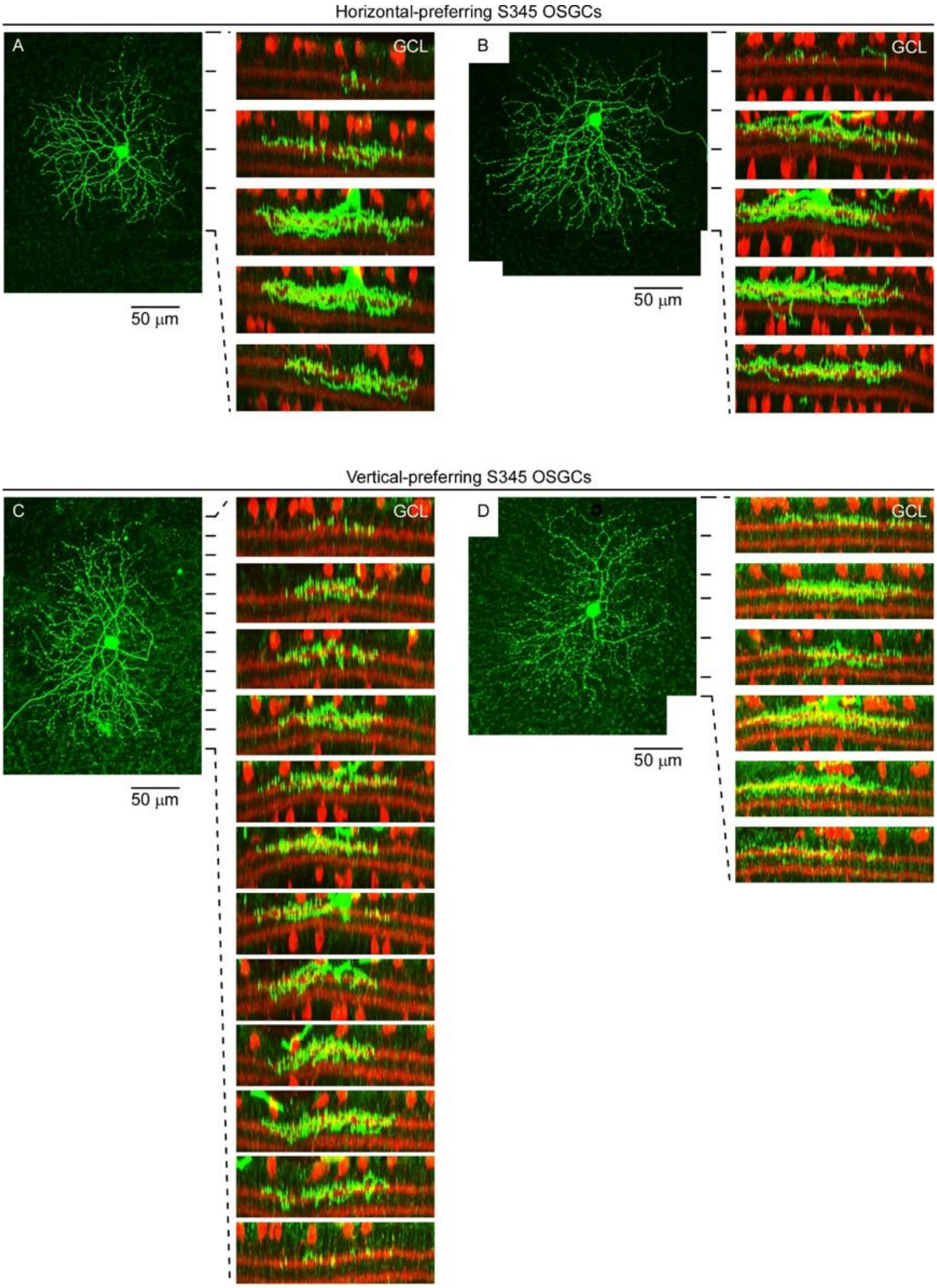

Figure S3. Representative morphology and dendritic stratification of S345 OSGCs. Related to figure 1.

(A, B) Representative morphology (left) and orthogonal view of dendritic stratification (right) of horizontal-preferring S345 OSGCs. Green, neurobiotin labeling of cells filled through the whole-cell recording pipette; red, ChAT immunostaining delineating the ON and OFF bands. The

dendrites of horizontal-preferring S345 OSGCs were concentrated primarily within IPL strata S3–S5, although a small number of dendritic terminals occasionally extended into strata S1–S2, as shown in (B). Scale bar, 50 µm.

(C, D) Representative morphology (left) and orthogonal view of dendritic stratification (right) of vertical-preferring S345 OSGCs. Green, neurobiotin labeling of cells filled through the whole-cell recording pipette; red, ChAT immunostaining delineating the ON and OFF bands. The dendrites of vertical-preferring S345 OSGCs were concentrated primarily within IPL strata S3–S5. Scale bar, 50 µm.

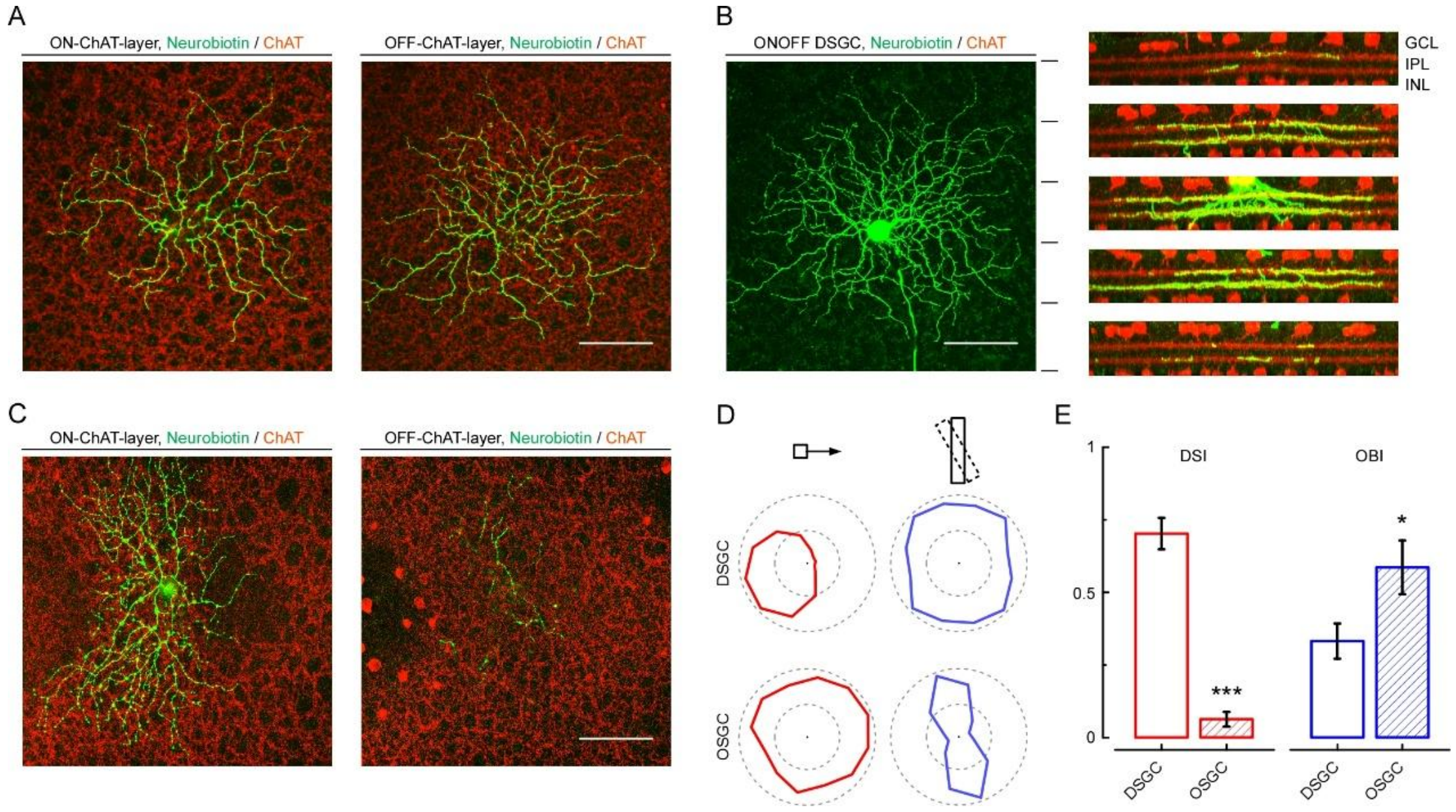

Figure S4: Assessing direction selectivity in OSGCs. Related to figure 1.

(A) Dendritic arbor (green) of the DSGC shown in (B), within the S4 sublayer (left, red: processes of ON starburst amacrine cells) and the S2 sublayer (right, red: processes of OFF starburst amacrine cells). Scale bar, 50 µm.

(B) Representative morphology (left, green) of an ON–OFF DSGC, and their dendritic stratification within the inner plexiform layer (right, green). ChAT immunostaining (red) delineates ON and OFF bands. Scale bar, 50 µm.

(C) Dendritic arbor (green) of the OSGC shown in (Fig. 1E-H), within the S4 sublayer (left, red: processes of ON starburst amacrine cells) and the S2 sublayer (right, red: processes of OFF starburst amacrine cells). Scale bar, 50 µm.

(D) Representative responses of a DSGC (upper) and an OSGC (lower) to moving square stimuli and bar stimuli presented along different orientations.

(E) Comparison of the direction selectivity index (DSI) and orientation bias index (OBI) between DSGCs and OSGCs (n = 15 DSGCs from 6 mice and 5 OSGCs from 5 mice). $p = 0.000002$ for DSI and $p = 0.0374$ for OBI; two-sided unpaired t-tests.

$^*p < 0.05$, $^{***}p < 0.001$. Data are shown as mean ± s.e.m.

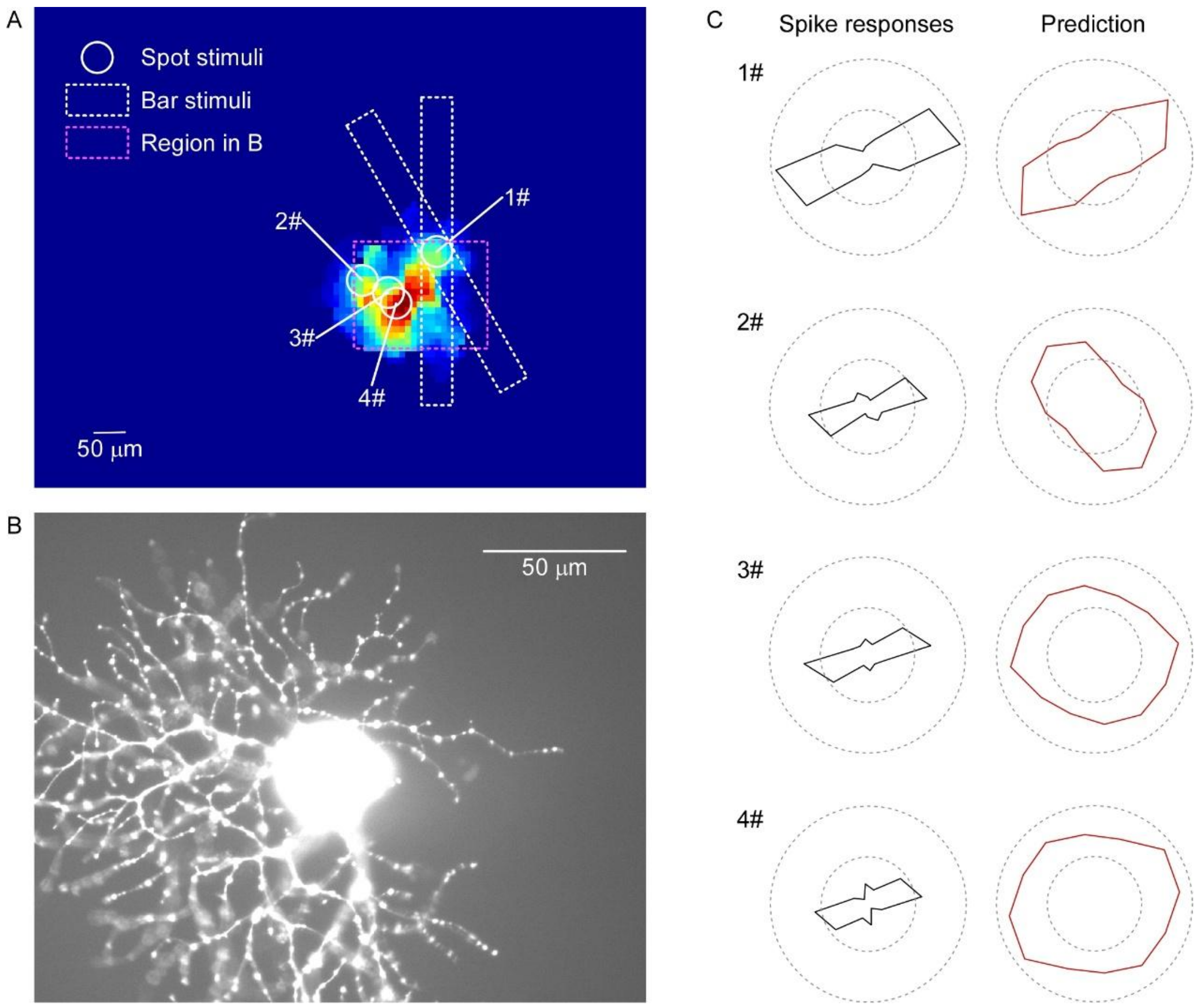

Figure S5: Mapping the receptive field of OSGCs. Related to figure 2.

(A) Receptive field center of an OSGC mapped with flashing spot stimuli (50 μm diameter). The excitatory center was derived from spike responses to stimuli presented at different spatial positions.

(B) Dendritic morphology of the same OSGC corresponding to the receptive field center shown in (A), displayed as a magnified view of the region outlined by the purple box.

(C) Polar plots (black) showing spiking responses of the OSGC to oriented bar stimuli presented at different positions within the receptive field, corresponding to the locations in (A). The predicted polar plot (red) represents the summed excitatory response to bars sweeping across the excitatory center.

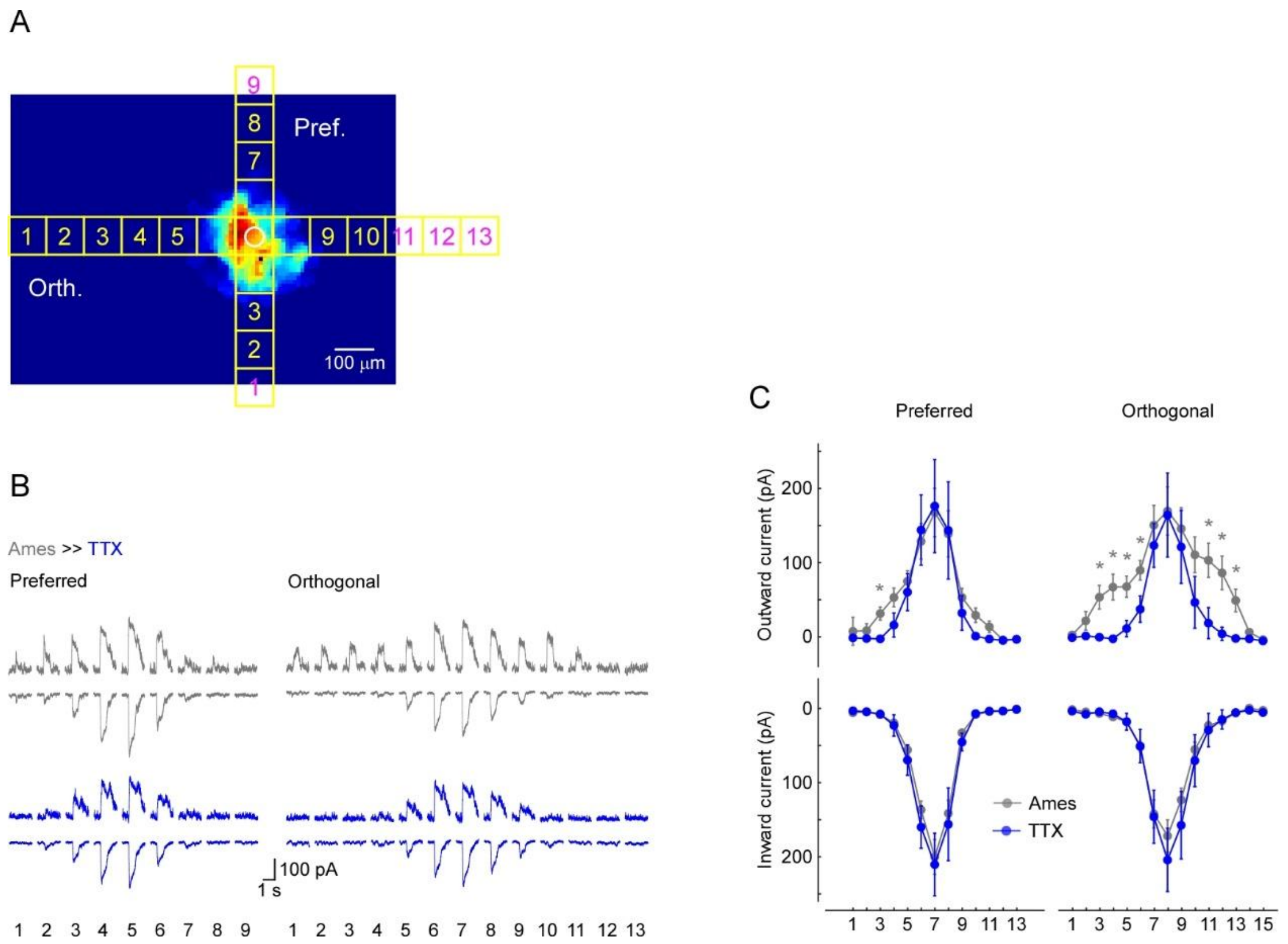

Figure S6. TTX suppresses distal inhibitory responses along the orthogonal axis of S345 OSGCs. Related to Figure 2.

(A, B) Square light stimuli (100 × 100 µm) were presented at different distances from the receptive-field center along the preferred and orthogonal axes of S345 OSGCs (A). Compared with the preferred axis, outward currents could be evoked at more distal locations along the orthogonal axis. These distally evoked outward currents were abolished after application of TTX (100 nM) (B). Panels A and B are rearranged versions of Figures 2A and 2B, respectively, with the traces displayed separately to avoid overlap and improve visibility.

(C) Effects of TTX on inward and outward currents evoked by square light stimuli presented at different distances from the receptive-field center along the preferred and orthogonal axes of S345 OSGCs. n = 10 and 4 cells for the Ames and TTX conditions, respectively, from a total of 10 mice. For outward currents along the preferred axis, gray asterisks indicate Ames versus TTX ($p = 0.0275$). Along the orthogonal axis, gray asterisks indicate Ames versus TTX (p = 0.0479, 0.0230, 0.0279, 0.0355, 0.0408, 0.0375, and 0.0494). Two-sided unpaired t-test . Panel C is a rearranged version of Figure 4M, with the response curves displayed separately to avoid overlap and improve visibility.

* $p < 0.05$. Data are shown as mean ± s.e.m.

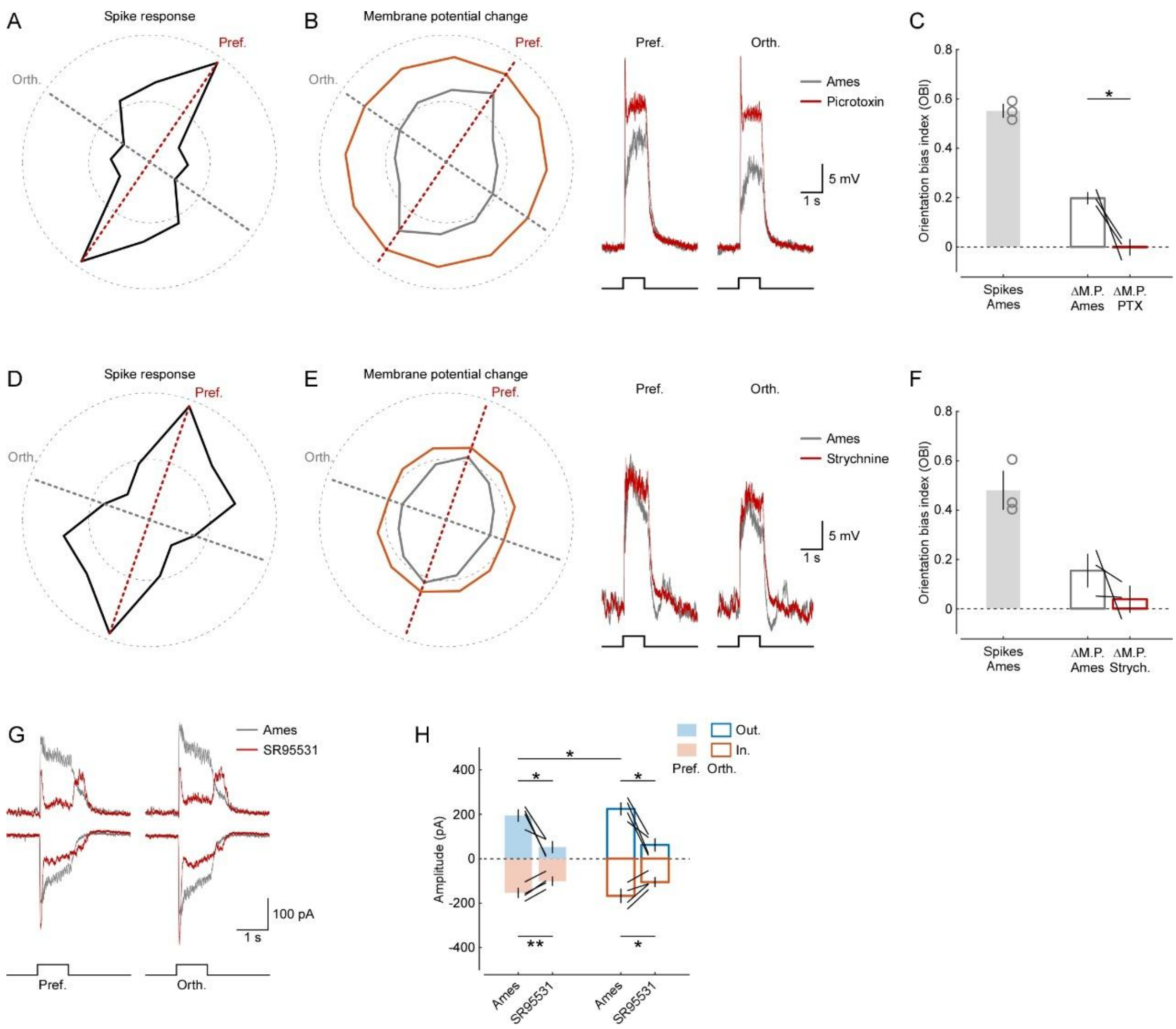

Figure S7: Effects of GABAergic and glycinergic inhibition on OSGC light responses. Related to figure 3.

(A, B) Spiking responses (A) and membrane potential changes (B) of an OSGC to bar stimuli presented at different orientations before and after application of picrotoxin (100 μM).

(C) Orientation bias index (OBI) of OSGCs before and after picrotoxin (PTX) application. n = 3 cells; p = 0.0433; two-sided paired t-test.

(D, E) Spiking responses (D) and membrane potential changes (E) of an OSGC to bar stimuli at different orientations before and after strychnine (2 μM) application.

(F) Orientation bias index (OBI) of OSGCs before and after strychnine (Strych.) application. n = 3 cells; two-sided paired t-test.

(G) Representative voltage-clamp recordings from an S345 OSGC in response to bar stimuli presented along the preferred (Pref.) and orthogonal (Orth.) orientations under control (Ames) and SR95531 (5 μM).

(H) Amplitudes of outward and inward currents evoked by bar stimuli along the preferred and

orthogonal orientations under control (Ames) and SR95531 conditions (n = 4 cells). p = 0.0282 (Pref. outward, Ames vs SR95531), 0.0165 (Orth. outward, Ames vs SR95531), 0.0010 (Pref. inward, Ames vs SR95531), 0.0173 (Orth. inward, Ames vs SR95531), and 0.0365 (Pref. vs Orth. outward, Ames); two-sided paired t-test.

* $p < 0.05$, ** $p < 0.01$, *** $p < 0.001$. Data are shown as mean ± s.e.m.

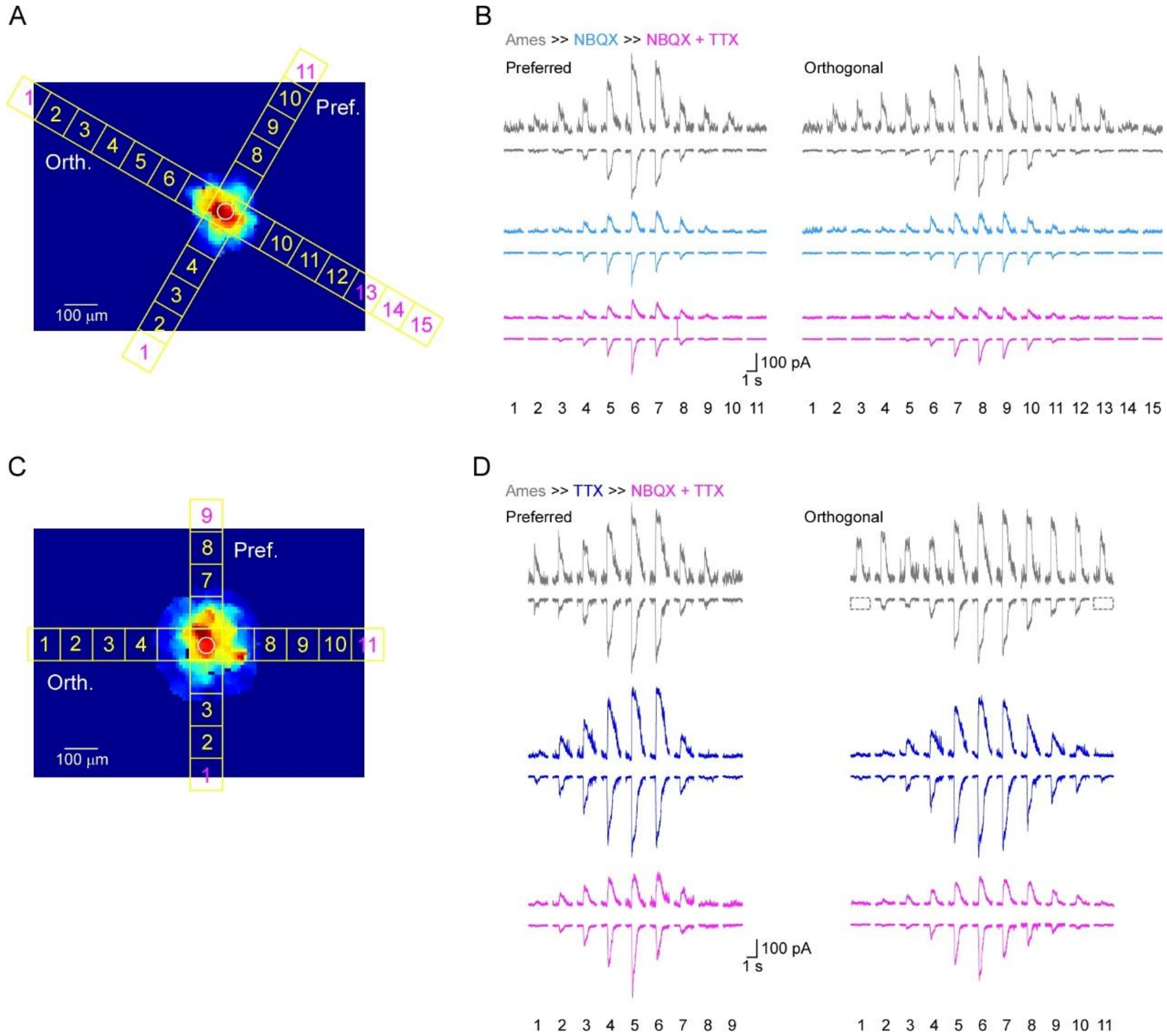

Figure S8. Effects of NBQX and TTX on spatially evoked current responses in S345 OSGCs. Related to Figure 4.

(A, B) Square light stimuli (100 × 100 µm) were presented at different distances from the receptive-field center along the preferred and orthogonal axes of S345 OSGCs (A), and the corresponding inward and outward currents were recorded under control (Ames), NBQX (5 µM), and NBQX (5 µM) + TTX (100 nM) conditions (B). Panels (A) and (B) are rearranged versions of Figures 4I and 4J, respectively, with the traces displayed separately to reduce overlap and improve visibility.

(C, D) Same as in (A) and (B), but recorded under control (Ames), TTX (100 nM), and NBQX (5 µM) + TTX (100 nM) conditions. Under the Ames condition, responses at the most distal stimulus positions were not recorded, as indicated by the dashed boxes. Panels (C) and (D) are rearranged versions of Figures 4K and 4L, respectively, with the traces displayed separately to reduce overlap and improve visibility.

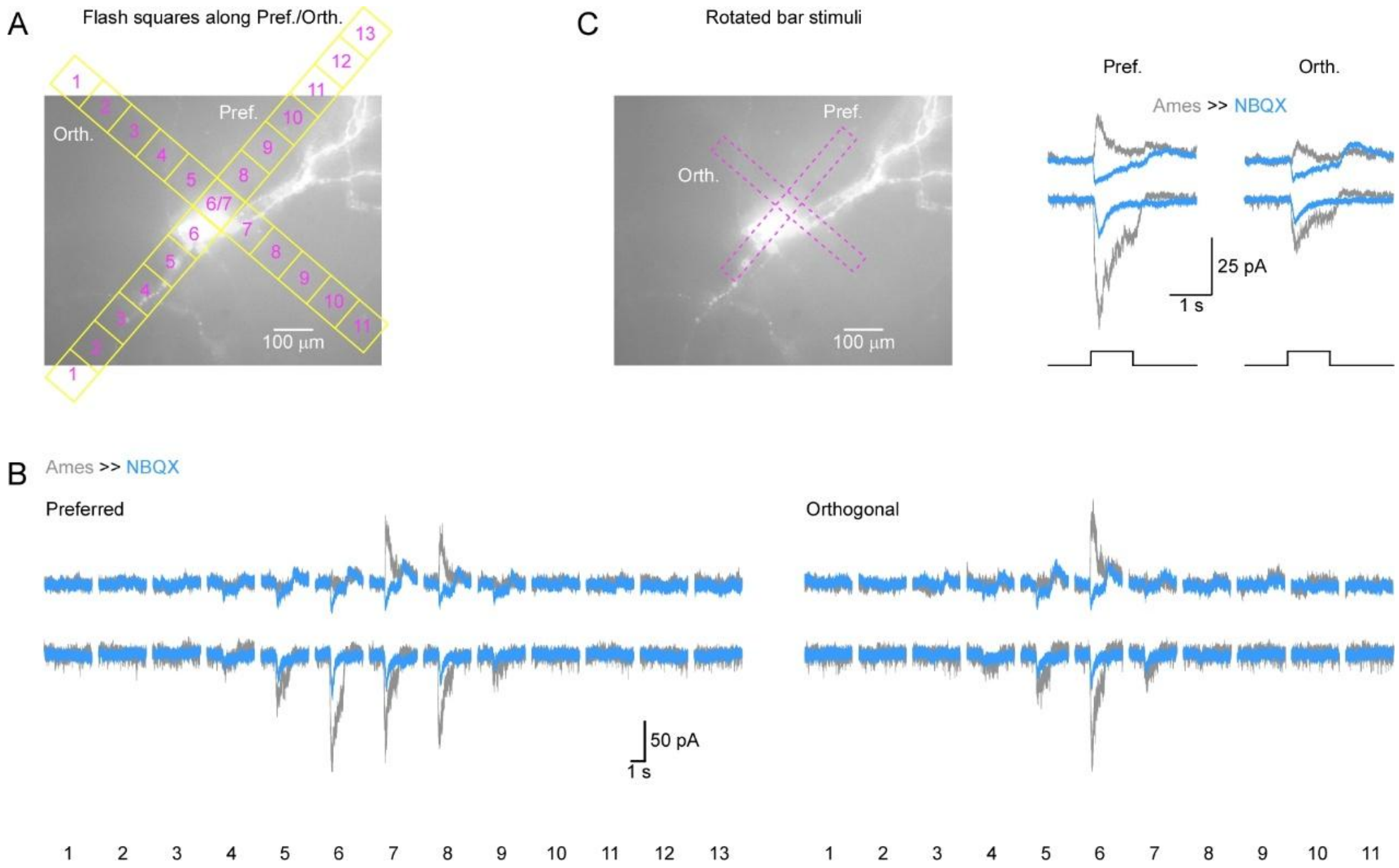

Figure S9: Effects of NBQX on OSAC light responses. Related to figure 5

(A) Representative experimental design showing flashing spot stimuli (100 × 100 μm) delivered at varying distances from the excitatory receptive field center of an OSAC along the preferred and orthogonal axes.

(B) Representative membrane current responses of the same OSAC to the stimuli shown in (A) under control (Ames) and NBQX (5 μM) conditions.

(C) Light responses of the OSAC evoked by bar stimuli centered on the excitatory receptive field (left) under control (Ames) and NBQX (5 μM) conditions.
